# Profiling genome‐wide methylation in two maples: Fine‐scale approaches to detection with nanopore technology

**DOI:** 10.1111/eva.13669

**Published:** 2024-04-17

**Authors:** Susan L. McEvoy, Patrick G. S. Grady, Nicole Pauloski, Rachel J. O'Neill, Jill L. Wegrzyn

**Affiliations:** ^1^ Department of Ecology and Evolutionary Biology University of Connecticut Storrs Connecticut USA; ^2^ Department of Forest Sciences University of Helsinki Helsinki Finland; ^3^ Department of Molecular and Cell Biology University of Connecticut Storrs Connecticut USA; ^4^ Institute for Systems Genomics University of Connecticut Storrs Connecticut USA

**Keywords:** *Acer negundo*, *Acer saccharum*, methylome, nanopore, nutrient stress, transposable elements

## Abstract

DNA methylation is critical to the regulation of transposable elements and gene expression and can play an important role in the adaptation of stress response mechanisms in plants. Traditional methods of methylation quantification rely on bisulfite conversion that can compromise accuracy. Recent advances in long‐read sequencing technologies allow for methylation detection in real time. The associated algorithms that interpret these modifications have evolved from strictly statistical approaches to Hidden Markov Models and, recently, deep learning approaches. Much of the existing software focuses on methylation in the CG context, but methylation in other contexts is important to quantify, as it is extensively leveraged in plants. Here, we present methylation profiles for two maple species across the full range of 5mC sequence contexts using Oxford Nanopore Technologies (ONT) long‐reads. Hybrid and reference‐guided assemblies were generated for two new *Acer* accessions: *Acer negundo* (box elder; 65x ONT and 111X Illumina) and *Acer saccharum* (sugar maple; 93x ONT and 148X Illumina). The ONT reads generated for these assemblies were re‐basecalled, and methylation detection was conducted in a custom pipeline with the published *Acer* references (PacBio assemblies) and hybrid assemblies reported herein to generate four epigenomes. Examination of the transposable element landscape revealed the dominance of *LTR Copia* elements and patterns of methylation associated with different classes of TEs. Methylation distributions were examined at high resolution across gene and repeat density and described within the broader angiosperm context, and more narrowly in the context of gene family dynamics and candidate nutrient stress genes.

## 1 INTRODUCTION

The processes shaping plant development and growth are regulated by epigenetic modifications that impact gene expression, genomic stability, and plasticity (Kumar & Mohapatra, 2021). Plant genomes are methylated in different ways at sequence contexts categorized as CG, CHG and CHH (where H = C, A, or T). These modifications are often initiated by transposable element (TE) activity, which impacts a significant portion of most plant genomes. Epigenetic modifications introduced through mobile elements contribute to genetic variation that is associated with biotic and abiotic stress adaptations (Ritter & Niederhuth, 2021).

Methylation in model plants has been studied from its initiation in embryos in *Arabidopsis thaliana* (Jullien et al., 2012), to the accumulation of methylation variation in the independent branches of a single *Populus trichocarpa* individual (Hofmeister et al., 2020). In *Arabidopsis*, the genome‐wide regulatory effects of methylation were demonstrated by the knockout of all five methyltransferases, impacting cell fate throughout the plant (He et al., 2022). Also in *Arabidopsis*, a heritable epiallele associated with climate was found to control leaf senescence, providing an example of local climate adaptation (He et al., 2018). In *P. trichocarpa*, epigenetic modifications were associated with changes in the circadian cycle (Liang et al., 2019). Increasingly, non‐model plant systems have been investigated, including for methylation responsible for leaf shape and photosynthetic traits in *Populus simonii* (Ci et al., 2016) and salinity‐induced methylation in mangroves (Miryeganeh et al., 2022).

Until recently, methylation was primarily investigated through treatments such as whole‐genome bisulfite conversion (WGBS). This technique is prone to degradation of DNA, incomplete conversion, and amplification bias (Gouil & Keniry, 2019). Since WGBS libraries are primarily short‐read sequenced, interpretation also suffers from poor resolution in repetitive regions. Long‐read technologies, including Pacific Biosciences' (PacBio's) single‐molecule real‐time (SMRT) sequencing, and nanopore sequencing by Oxford Nanopore Technologies (ONT), can detect methylation. SMRT sequencing can detect 5mC modifications based on polymerase dynamics at very high coverage, as well as methods that rely on bisulfite conversion for standard coverage. In comparison, nanopore sequencing can directly detect DNA or RNA modifications through a voltage‐measured pore, enabling real‐time, single‐molecule sensitivity (Liu et al., 2021).

Being sessile, plants rely on methylation as an evolutionary strategy in the form of short‐ and long‐term (heritable) stress memory (Sun et al., 2022), and this is particularly important in long‐lived tree species with slower changes in allele frequency (Bräutigam et al., 2013). DNA methylation is known to have a critical role in the silencing of TEs, but environmental stress can reduce this activity and result in TE bursts (Cavrak et al., 2014). Epigenetic mechanisms related to transposition have been associated with responses to drought, temperature, and nutrient stress (Fan et al., 2022). In the context of maples, sugar maple (*Acer saccharum*) is susceptible to calcium deficiency, and this has led to a significant decline in natural populations (Bishop et al., 2015). The first comparative genomics study on North American maples identified candidate genes from the analysis of expression in the aluminum‐ and calcium‐amended plots at the Hubbard Brook Experimental Forest (McEvoy et al., 2021). The interplay between TEs and gene expression is complex, but many forest tree species would benefit from a deeper examination to fully understand their adaptive potential for new and ongoing threats.

This study extends the previous work on *Acer negundo* and *A. saccharum* (McEvoy et al., 2021), as well as that of Sork et al. (2022) and Niederhuth et al. (2016) in comparative plant methylomics. Employing ONT sequencing from two previously unstudied *Ace*r individuals, we completed genome assembly and annotation and detected methylation sites genome‐wide. We customized methylation calling methods, and in doing so, generated comparative methylation profiles focusing on transposable elements and nutrient stress candidate genes.

## 2 METHODS

### 2.1 Sequencing

Leaves from two maple individuals, *A. negundo* (Accession 253‐2013*B) and *A. saccharum* (1353‐84*A), were shipped on dry ice from the Arnold Arboretum of Harvard University. High molecular weight (HMW) DNA was extracted from both samples using the ONT protocol for *Arabidopsis* leaves (Vaillancourt & Buell, 2019). The resulting gDNA was checked for quality control via Thermo Scientific Nanodrop and then Agilent TapeStation (Agilent Technologies, Santa Clara, CA). Libraries were prepared with the ONT Genomic DNA by Ligation protocol with additional Covaris shearing to improve coverage (Oxford Nanopore Technologies, Oxford, UK). A single ONT Flongle sequencing run was conducted to evaluate the library quality prior to the PromethION run (*A. negundo*: 15 K reads, N50 16 Kb in 23 hours; *A. saccharum*: 39 K reads, N50 14 Kb after 21 hours). Two PromethION runs (one per individual) followed, using flow cell type FLO‐PRO002, kit SQK‐LSK110, and Guppy v4.0.11 (Oxford Nanopore Technologies, 2020a) with the high‐accuracy basecalling model and read filtering minimum qscore of 7. To reduce the error rate, particularly for methylation calling, the resulting FAST5s were re‐basecalled with Guppy v5.0.16 (GPU) and ONT super accuracy model r9.4.1_450bps_sup. The same samples were short‐read sequenced in a single Illumina NovaSeq 6000 SP v1.5 300 cycle run. TruSeq DNA Nano with Covaris shearing was used for Illumina library preparation in advance (Illumina Inc., San Diego, CA, USA).

### 2.2 Assembly and annotation

Illumina short reads were quality‐controlled with FASTP v0.22.0 (Chen et al., 2018). ONT R9 long‐reads were filtered for archaea, bacteria, fungi, and virus contaminants via Centrifuge v1.0.4‐beta (Kim et al., 2016) as well as length (5 Kb minimum) using NanoPlot v1.33.0 (De Coster & Rademakers, 2023). The filtered ONT reads were combined with raw Illumina reads for hybrid assembly using MaSuRCA v4.0.3 (Zimin et al., 2017). This assembly contained minimal duplication and attempts to remove redundant haplotypes reduced overall completeness, so this step was eliminated, aiming for a consensus haploid assembly. Short‐read polishing was conducted with Pilon v1.24 (Walker et al., 2014) using the trimmed short reads that were aligned with Bowtie v2.3.5.1 (Langmead et al., 2009). Scaffolding was performed with RagTag v2.1.0 (Alonge et al., 2021) using the original chromosome‐scale reference genomes for the same species (McEvoy et al., 2021).

Repeats were identified with RepeatModeler v2.0.1 (Flynn et al., 2020) and combined with the LTR reference library InpactorDB non‐redundant v5 (Orozco‐Arias et al., 2021) for softmasking with RepeatMasker v4.0.6 (Smit et al., 2013–2015). ParseRM (Kapusta, 2017) generated repeat summaries and estimates of abundance by divergence. BRAKER2 v.2.1.6 predicted genes with the previously published RNA‐Seq leaf tissue library provided as evidence (Brůna et al., 2021) The RNA‐Seq leaf tissue libraries represented 31.2 M read pairs for *A. negundo* and 30.6 M read pairs for *A. saccharum*. Gene models were filtered with gFACs v1.1.3 (Caballero & Wegrzyn, 2019) with the following: unique genes only, mono‐exonics missing a start or stop codon or valid protein domain, mult‐iexonics missing both a start and stop codon, and genes with exons smaller than 6 bp. Functional annotation of the final gene space was conducted with EnTAP v0.10.7 using RefSeq Complete and Uniprot NCBI databases, along with Eggnog v4.1 for gene family assignment (Hart et al., 2018; O'Leary et al., 2016; UniProt Consortium, 2019).

### 2.3 Methylation detection

The METEORE pipeline was selected as the general approach to methylation detection based on its ability to generate reliable consensus results from multiple tools (Yuen et al., 2021). Two tools were selected: Nanopolish v0.13.2 (Loman et al., 2015) and DeepSignal‐Plant v0.1.4 (Ni et al., 2021). This pairing was chosen as it had favorable results in the Yuen et al. (2021) benchmarking study and the ability to inform beyond the CG‐context. Nanopolish is a well‐supported tool that detects methylation in the CG context. DeepSignal‐Plant is the top‐performing, most accessible tool trained with plant‐based models to detect methylation in CHG and CHH contexts from nanopore sequencing (Ni et al., 2021).

**FIGURE 1:**
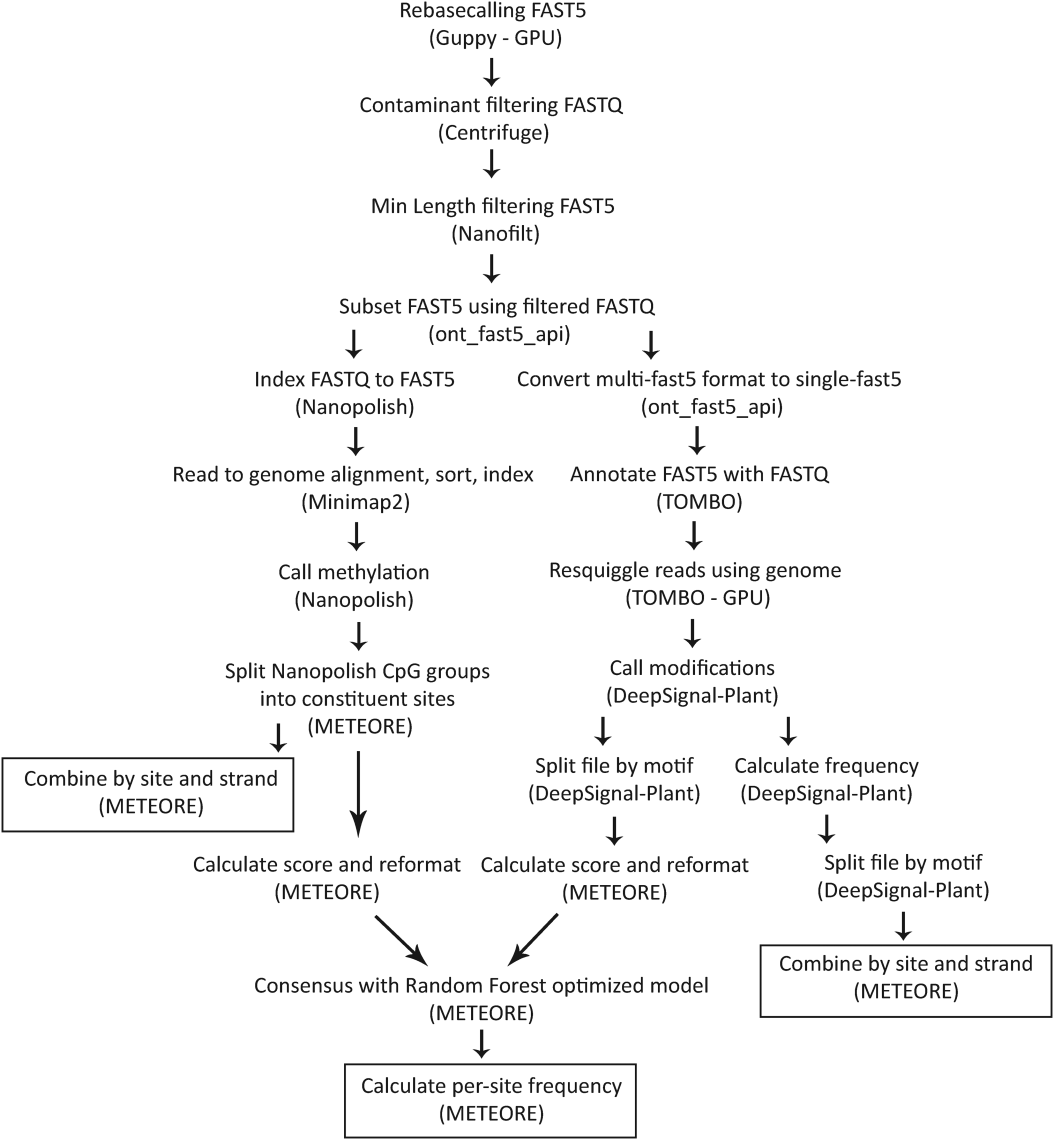
Stepwise method of methylation detection using the METEORE pipeline to create a consensus of DeepSignal‐Plant and Nanopolish.

ONT reads from the new individuals were aligned to both the new and original genomes for methylation calling. Methods for Nanopolish and DeepSignal‐Plant proceeded according to the documentation for each tool and the Snakemake workflows provided by METEORE (Figure 1). Each tool was run to create two output formats: (1) the input format necessary for integration in the consensus, and (2) the standardized form of independent tool output, calculating per‐site and per‐strand frequencies, allowing for greater ease of interpretation across tools. This provided both tool‐specific frequencies and a consensus for comparison.

To begin, the re‐basecalled and filtered FASTQ reads were used to filter the FAST5 using ont_fast5_api ‘fast5_subset.py’ v4.0.0 (Oxford Nanopore Technologies, 2021). Nanopolish requires indexing of FASTQ to FAST5 in the default multi‐fast5 format, and then alignment with minimap2 with parameters ‐ax map‐ont (Li, 2018). These alignments were run with minimap2 version 2.22‐r1101 outside of the METEORE pipeline because the within‐METEORE version provided by Snakemake was older. The next step was Nanopolish ‘call_mods’, followed by METEORE scripts to convert the output of log‐likelihood ratio values into a standardized format.

DeepSignal requires single‐fast5 formatted files, so the multi‐fast5 files were converted using ont_fast5_api ‘multi_to_single_fast5’. DeepSignal‐Plant began with annotation of the FAST5s with FASTQ using Tombo ‘preprocess annotate_raw_with_fastqs’ v1.5.1 (Oxford Nanopore Technologies, 2020b). This was followed by Tombo ‘resquiggle’ with the parameter –signal‐align‐parameters 4.2 4.22000 2500 20.0407502500250 used for the original genomes only. It is possible to detect methylation on either reads or extracted features; reads were recommended, so this method was implemented. At this stage, results were split into separate files for CG, CHG, and CHH subcontexts by modifying METEORE split_freq_file_by_5mC_motif_sm.py. METEORE scripts were then used to standardize per‐site and per‐strand formats as described for Nanopolish. The CG file alone was formatted as input for the consensus script. Consensus predictions were generated using a Random Forest with provided models optimized at n‐estimator = 3 and max_dep = 10. Results were analyzed with BEDTools v2.29.0 (Quinlan & Hall, 2010) and karyoplotR (Gel & Serra, 2017).

### 2.4 Statistical analysis and visualization

BEDTools v2.29.0 (Quinlan & Hall, 2010) was used to create 1 Mb windows and 100 Kb windows with 50 Kb overlaps across the genomes. It was then used to map methylation to these windows and calculate the frequency mean. BEDTools was also used with gene annotation files to intersect the gene regions and count methylated sites within the region. Plotting of chromosomal distributions was conducted with karyoplotR v1.21.3 (Gel & Serra, 2017). Pearson correlation was calculated with R Core Stats Package v4.2.0 (R Core Team, 2013). Statistics and summaries were used to compare across reference genomes within *Acer* (McEvoy et al., 2021), as well as *Populus* (Hofmeister et al., 2020), *Quercus* (Sork et al., 2022), and the 34 angiosperms surveyed by Niederhuth et al. (2016). Rapidly expanding and contracting families within *A. negundo* and *A. saccharum* were plotted along the distributions of methylation frequency and TE coverage. A total of 245 candidate genes associated with calcium response in *A. saccharum* were also investigated.

Scripts for all methods are available at doi: 10.5281/zenodo.10659005.

## 3 RESULTS AND DISCUSSION

### 3.1 Sequencing

The ONT sequencing of *A. negundo* resulted in 48.5 Gb (115x coverage) in 4.29 M reads with a read N50 of 16.3 Kb. Re‐basecalling resulted in an expected loss, resulting in 29.6 Gb (70x) in 2.55 M reads with an N50 of 16.2 Kb. After contaminant (56 K reads) and length filtering, 65x coverage with an N50 of 16.6 Kb remained. Illumina raw reads had 111x coverage as 46.6 Gb in 310 M reads. After trimming for adaptors, length, and quality, 104x coverage (146 M total read pairs) remained (File S1). Nanopore sequencing of *A. saccharum* resulted in 104.5 Gb in 9.8 M reads with an N50 of 15.1 Kb. Re‐basecalling resulted in 56.7 Gb (99x coverage) in 5.2 M reads with an N50 of 15.2 Kb. Filtering (135 K contaminant reads) generated coverage of 93x with a read N50 of 15.5 Kb. Illumina sequencing (150 bp PE) produced 148x coverage of 52.9 Gb bases in 563 M read pairs and trimming reduced this to 139x (526 M total read pairs).

### 3.2 Assembly and annotation

After the initial draft assembly, *A. negundo* had a total length of 421 Mbp in 421 contigs and an N50 of 2.16 Mb. BUSCO embryophyta genes were 96.0% complete, with 2.9% of these in duplicate. Polishing only minimally reduced the length and N50 (File S1). Scaffolding with the original genome increased the length slightly, but it remained within 421 Mbp, and the N50 grew to 33 Mb. The assembly was in 32 scaffolds, with 13 chromosomes representing 99.7% of the assembled length. The genome size remained constant, though slightly smaller, than the published reference (442 Mbp) and closer to the kmer‐based estimate of 319 Mbp (McEvoy et al., 2021). At 96%, the final complete assembly BUSCO scores remained similar to the draft, with duplicates dropping slightly to 2.8%, 0.7% fragmented, and 3.3% missing (Table 1, File S1). The lower duplicate value was an improvement from the original reference duplication of 3%, although the completed single copy dropped slightly. Structural annotation of the new genome identified 27,541 genes, of which 23,408 were functionally annotated by either similarity search or gene family assignment. The BUSCO score for annotated proteins was 92.1% complete, with 3% in duplicates (Table 1).

**TABLE 1:** Assembly and annotation statistics for new and original genomes.

Assembly	Genome			Genes				
	Length	N50	BUSCO^a^	Total	Mono‐ exonic	Average length	Avg. CDS length	BUSCO^a^
*Acer negundo* v2	421 Mbp	33 Mb	96.0 (2.8)	27,541	3474	3098	1122	92.1 (3.0)
*A. negundo* v1	442 Mbp	32 Mb	97.7 (3.0)	30,491	5558	5386	1174	94.1 (6.8)
*Acer saccharum* v2	571 Mbp	41 Mb	96.2 (6.5)	35,834	5366	2918	1087	91.8 (8.5)
*A. saccharum* v1	626 Mbp	46 Mb	97.4 (5.6)	40,074	8765	6761	1190	93.1 (7.7)

^a^ Complete % (Duplicate %), Embryophyta 10.

The *A. saccharum* draft assembly had a total length of 571 Mbp in 1194 contigs and an N50 of 805 Kb. BUSCO embryophyta genes were 96.0% complete, with 6.6% of those in duplicate. Similar to *A. negundo*, polishing slightly reduced the total length and N50 in this genome. The BUSCO duplicate score increased by 0.1%. The BUSCO of the scaffolded genome was 96.2% complete, with 6.5% in duplicate, 0.7% fragmented, and 3.1% missing. The assembly had a total length of 571 Mbp in 160 contigs and an N50 of 41 Mb (Table 1, File S1). Scaffolding resulted in 13 chromosomes that represented 96.8% of the assembled length. The genome size dropped from the published reference of 626 Mbp, a more substantial decrease than seen in *A. negundo*, and below the original kmer‐based estimate of 636 Mbp. A total of 35,834 genes were identified, and 29,858 were associated with functional information. The resulting annotation BUSCO score was 91.8% complete, containing 8.5% duplicates (Table 1, File S1).

**FIGURE 2:**
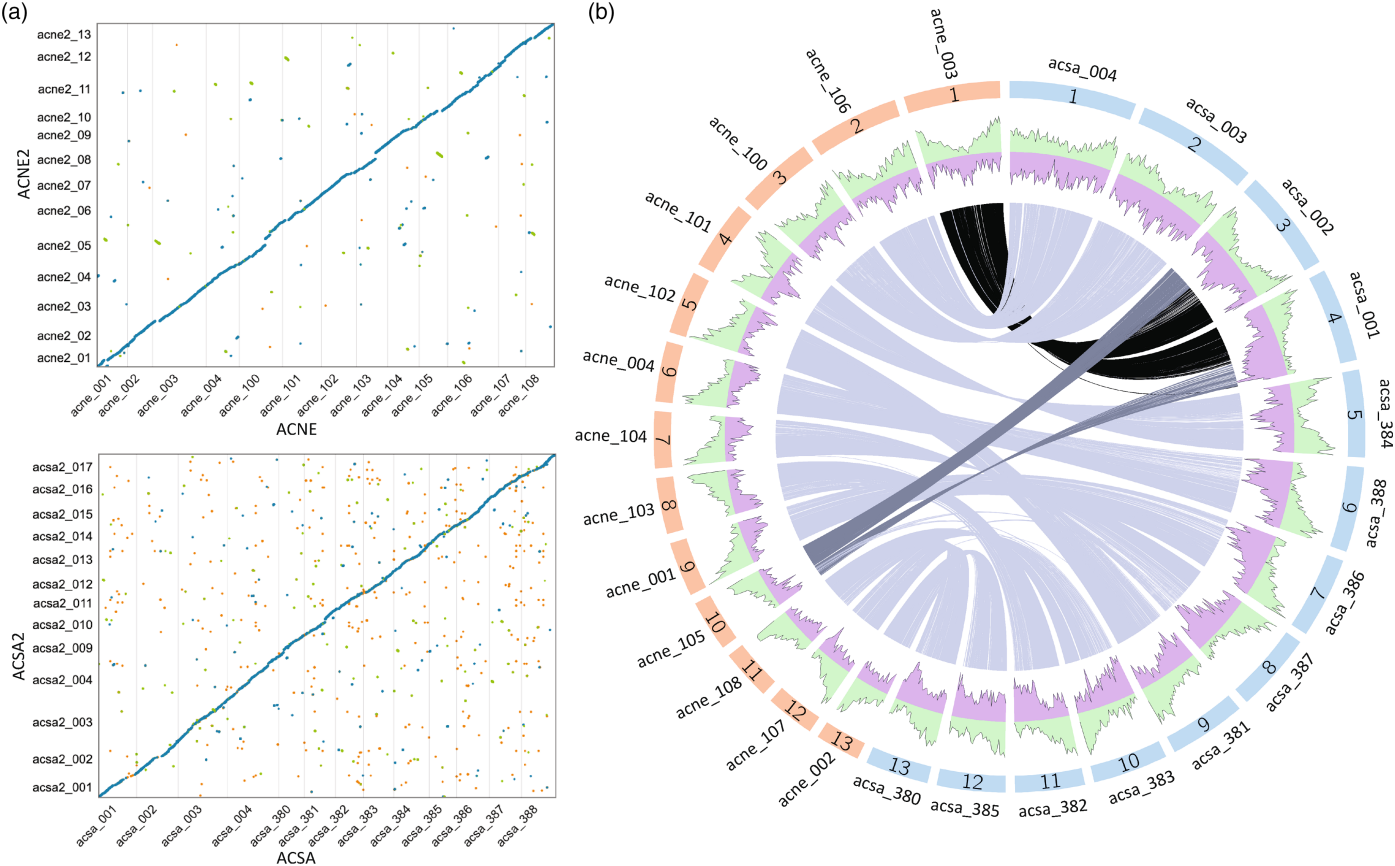
(a) Alignment of reference genomes comparing original (x‐axis) and new (y‐axis) assemblies for *Acer negundo* (ACNE) and *Acer saccharum* (ACSA). Blue indicates forward alignments, green indicates reverse, and orange is repeated alignments. (b) Gray lines show blocks of syntenic genes between *A. negundo* (orange) and *Acer saccharum* (blue) using the original reference genomes (reprinted from McEvoy et al. (2021)). The green area depicts gene density and purple is repeat frequency.

The first versions of the *Acer* genomes were produced exclusively with deep‐coverage PacBio reads (PacBio Sequel II, >100X), and resulted in fairly contiguous references that assembled to chromosome‐scale with the addition of HiC libraries (McEvoy et al., 2021). The two new accessions, assembled in a hybrid manner and scaffolded with the published PacBio references as described above, were smaller than the original references (Table 1). The difference in size could be structural variation between the different genotypes, but more likely reflects some of the differences in read inputs and methodology (i.e. assembler). *A. negundo* was most similar to its original genome, with an improved duplication rate, and the missing genes identified (~3 K) were not specific to the new genome. *A. saccharum* had an increase in BUSCO‐estimated duplication and 4 K original genes were not observed in the new assembly. Similar to the assembly, differences in final gene number may partially reflect variation in the BRAKER software used for prediction. Whole‐genome alignment between the new and original versions revealed that, of the existing assembled sequence, no major discrepancies were present in either species (Figure 2a). Links between syntenic regions of the original genomes in both species showed similarities in spite of the larger genome size of *A. saccharum* (Figure 2b).

### 3.3 Methylation within *Acer*

Long‐read sequencing for whole‐genome methylation detection and quantification has not yet been widely adopted in plants. *Brassica nigra* was among the first plants on which such analyses were conducted, with ONT reads enabling the demarcation of the centromeres (Perumal et al., 2020); an improved nanopore‐sequenced radish genome provided the same centromere‐level resolution (Cho et al., 2022). Both centromeres and stress responses were studied in *Gossypium thurberi* and *Gossypium davidsonii* through 5mC and 6 mA sites (Yang et al., 2021). The first release of DeepSignal was used with ONT reads to detect novel repeats and characterize the subtelomeres in algae (Chaux‐Jukic et al., 2021).

The DeepSignal‐Plant tool utilized ONT data from *A. thaliana, Oryza sativa*, and *B. nigra* for development and benchmarking (Ni et al., 2021). This tool represents the first machine‐learning approach for plants that can achieve accuracy for all three important 5mC states: CG, CHG, and CHH. The potential roles of methylation in each context are currently being studied in model and non‐model systems, but it appears that non‐CG methylation can have a stronger effect in silencing TEs, and CHH is responsible for regulating both CG‐ and CHG‐modified TEs (Domb et al., 2020; Ni et al., 2021). Studies on transgenerational inheritance of these forms of 5mC modifications in *Arabidopsis* determined that asymmetric CHH must be re‐established de novo, while symmetric CG and CHG methylation is maintained (Hsieh, 2016). Using the consensus‐based approach from METEORE, we combined DeepSignal‐Plant with one of the best implementations of an HMM‐based approach, Nanopolish, leveraging the strengths of both to detect methylation across all contexts in ONT sequencing (Yuen et al., 2021).

**FIGURE 3:**
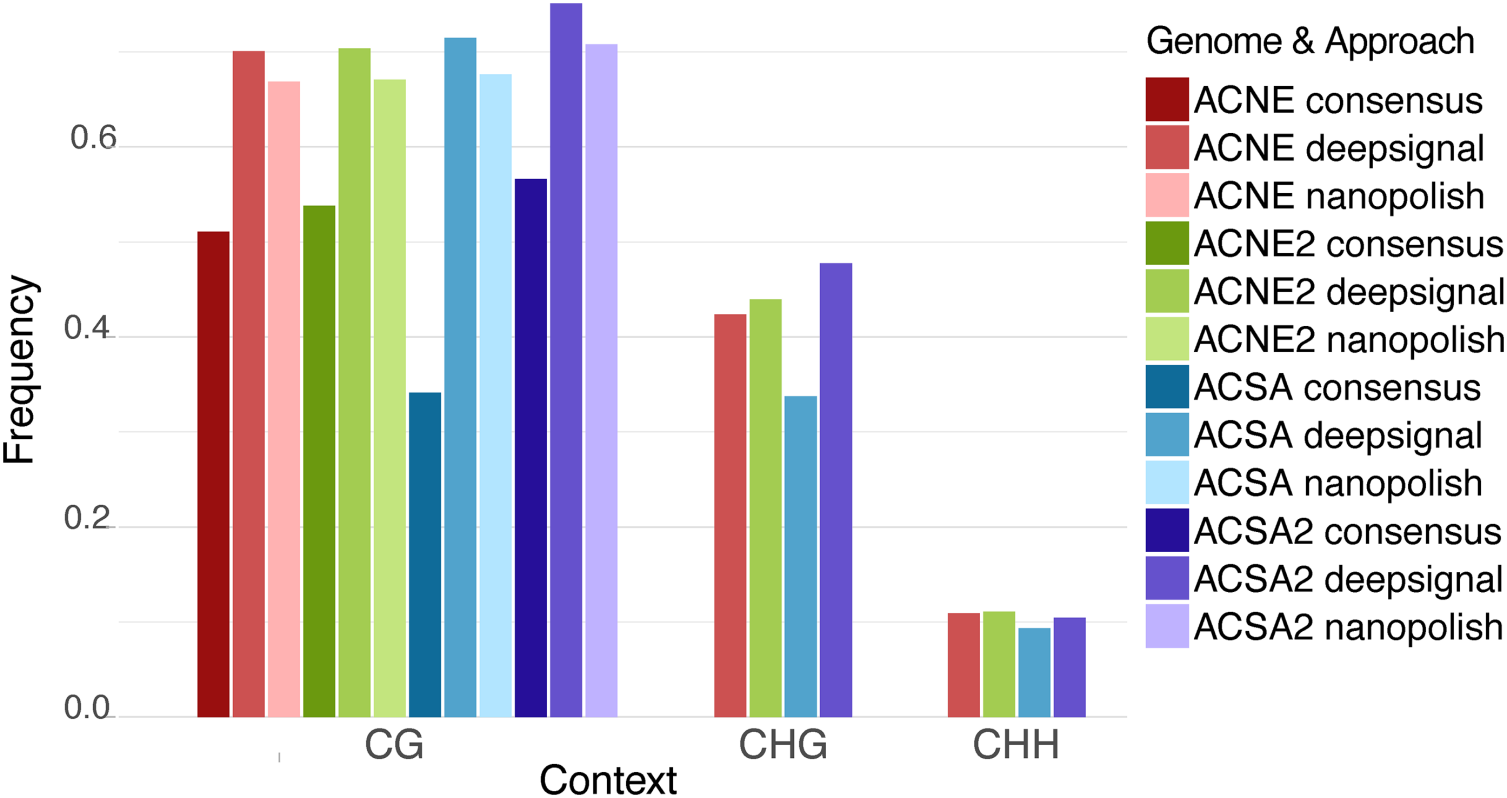
Comparison of global methylation levels by context for DeepSignal‐Plant, Nanopolish, and the METEORE‐generated random forest consensus.

Independently assessed methylation levels for CG sites reported by Nanopolish and DeepSignal‐Plant were both high relative to the consensus (Figure 3). DeepSignal‐Plant detected the most CG‐methylation across all four genomes, as was seen in benchmarking studies comparing DeepSignal (v1) and Nanopolish on *E. coli* and *H. sapiens* data (Ni et al., 2019). The CG levels for both the new and original *A. negundo* and original *A. saccharum* were quite similar (~70%), and the new *A. saccharum* genome was higher, at 75%. Nanopolish results followed a similar trend, but with slightly lower values of ~67%, and 71% for the new *A. saccharum* genome. Consensus results for all were around 53%. In particular, the original *A. saccharum* genome was considerably lower, at 34%—close to the CHG value, which was unexpected. The reduced consensus reported for all genomes is likely a result of METEORE's ability to resolve false positives. Benchmarking studies found that when used independently, tools such as Nanopolish and Deepsignal tend to overpredict in certain situations, such as regions of hypomethylation (Yuen et al., 2021). METEORE uses read scoring and random forest methods to more accurately predict consensus results including removal of false positives. Results presented here would best tested by including additional tools with different limitations.

The CHG values estimated by DeepSignal‐Plant were in the 40% range but, again, the original *A. saccharum* genome value was lower than those of the other genomes, at 33%. CHH values were the lowest, and more consistent across all four, ranging from 9% to 11%.

The original *A. saccharum* genome resulted in much lower estimates of methylation compared to the other three genomes. This genome had the lowest retention of sequences during resquiggling, a preliminary step for DeepSignal‐Plant that leverages the reference genome to correct base calling inaccuracies (Oxford Nanopore Technologies, 2020b). When resquiggling the reads to the original and new *A. negundo* genomes, 21.2% and 18.0% reads were unsuccessfully processed. For *A. saccharum* these numbers were 54.3% and 31.5%, resulting in 43x coverage for the original genome (File S1). Filtering was largely due to poor raw to expected signal matching (35.7%). This error might indicate significant discrepancy between the original *A. saccharum* genome and the new ONT which is reflected in assembly statistics reported in Table 1. Perhaps poor signal alignment and/or lower data coverage could have impacted detection in Nanopore or DeepSignal‐Plant thus reducing METEORE's random forest‐generated consensus (Figure 3).

Given the reduced CG methylation levels of the consensus derived from the original *A. saccharum* genome, the methylation datasets based on the new genomes were selected as the best representation. As such, these genomes were used for downstream analyses conducted with the METEORE consensus results for CG methylation and DeepSignal‐Plant results for CHG and CHH where no Nanopolish results exist. Figures displaying results for both new and original genomes are available in the supplementary material (Figures [Link], [Link], [Link], [Link], [Link]).

### 3.4 Comparative methylomes

Independent estimates from DeepSignal‐Plant and Nanopolish were on the higher end of the spectrum of global methylation levels reported for 34 angiosperms in Niederhuth et al. (2016). Consensus results were closer, but still higher than many angiosperms, and similar in total to *Fragaria vesca*, *Manihot esculenta*, *Vitis vinifera*, *Brachypodium distachyon*, and *Setaria viridis*. WGBS in the form of MethylC‐Seq was used in the Niederhuth et al. (2016) study, so increased signal detection could result from the long‐reads, which are able to span low‐complexity regions. A recent study compared short‐read and long‐read assemblies of the *Brassica* genome with both WGBS and ONT‐based approaches (Nanopolish). The direct CG methylation profiling using the ONT reads was strongly correlated (>93%) with their WGBS data for the same accession. In addition, ONT long‐reads identified centromeric repeats and accompanying hypermethylated signal (Perumal et al., 2020).

**FIGURE 4:**
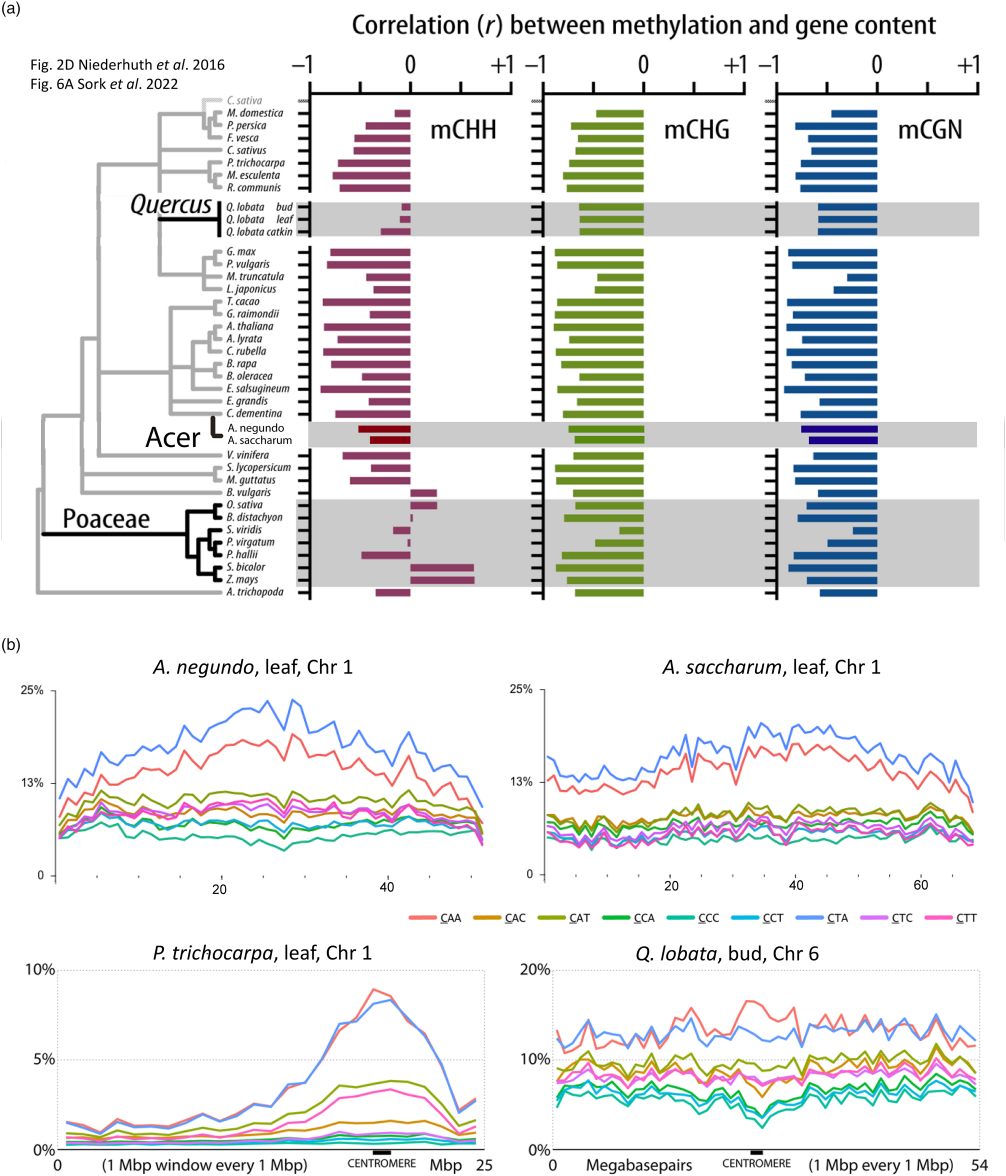
(a) Modified from Sork et al. (2022) to add the *Acer* genomes. *Acer* has a negative correlation between methylation and gene content, similar to other species. In contrast, *Quercus* or *Poaceae* are less negatively correlated, or even positively correlated in some species. (b) Distribution of CHH methylation by subcontext across the longest chromosomes for *Acer negundo* and *Acer saccharum*. *Populus* and *Quercus lobata*, reprinted from Sork et al. (2022), are shown for comparison of distribution patterns, where *Populus* is localized and *Q. lobata* is generally distributed. Distributions for all chromosomes are in Figure S1. CHG distributions are in Figure S2 and gene densities plotted with all contexts in 100 Kb windows are in Figure S3.

The distribution of methylation, genes, and TEs is of interest due to the implications for evolutionary strategies involving methylation's regulation of genes, TEs, and the boundary regions between. Many plant species exhibit a pattern of gene density along the arms of the chromosome and TE density in centromeric regions (Comai et al., 2017). As an exception, Sork et al. (2022) demonstrated that *Quercus lobata* has a more uniform distribution of genes and CHH methylation along the chromosome arm, similar to several *Poaceae*. The Pearson correlation (*R*) of genes to methylation for each sequence context was plotted along with the previous analyses and a rudimentary phylogeny (Niederhuth et al., 2016; Sork et al., 2022) (Figure 4a). In *Acer*, which is evolutionarily diverged from *Quercus*, there is a moderate negative correlation between CHH methylation and genes—almost 50%—similar to about half of angiosperms surveyed, while the remaining angiosperm species have stronger negative correlation (~75%). CHH was the context with least negative correlation of genes to methylation for *Acer*. Less correlation of methylation with genes means more intersection in TE filled intergenic space, lending support to the hypothesis that in some species, the balance of CHH methylation, TE suppression, TE proximity to genes, and gene body methylation could indicate complex strategies playing a role regulating key genes at TE boundary regions, as observed in grasses (Martin et al., 2021) and *Oryza* (Gallo‐Franco et al., 2022).

Previous analysis of CHH subcontexts across chromosomes detected differences between *Q. lobata*, in which CHH is broadly distributed, and *Populus trichocarpa*, in which CHH is localized around the centromere and decreases across the gene‐dense chromosome arms (Sork et al., 2022). *Acer* presents an intermediate pattern, particularly *A. saccharum* (Figures 4b and S1). *A. saccharum* does not have the same observable enrichment of CHH adjacent to centromeres as *P. trichocarpa*, nor is CHH as generally distributed as in *Q. lobata*. In both *Acer* species, there is a clear pattern for a preference of CTA, followed by CAA, along the lengths of the chromosome arms. Cytosine methyltransferases (CMT) are known to have sequence context preferences. In *Arabidopsis*, CMT2 prefers CHH sites, specifically CAA and CTA. However, little is known about these preferences outside of a handful of model species (Raju et al., 2019).

### 3.5 Repeats

LTR superfamilies *Ty1‐copia* and *Ty3‐gypsy* typically contribute the greatest proportion of TEs in land plants. Insertions of LTRs in and around genes can be responsible for alternative splicing, duplication, recombination, and epigenetic control (Galindo‐González et al., 2017). Biotic and abiotic stress, as well as other external stimuli, can lead to TE movement and the rapid proliferation of several subfamilies and may result in polyploidization (Mhiri et al., 2022). The exact mechanisms of activation and repression are not fully understood, but generally involve methylation in any of three sequence contexts. Activation of TEs leads to an initial response of post‐transcriptional gene silencing involving siRNA, which is followed by establishment and maintenance of silencing with DNA and histone methylation in RNA‐directed DNA methylation (RdDM) pathways. RdDM methylation leads to chromatin modification making the TE inaccessible to transcriptional machinery (Erdmann & Picard, 2020). The intersection of methylation and TEs can be informative for understanding control of expression and architectural changes to the genome that may play a role in evolution.

Repeat detection and classification with the LTR database, in addition to de novo libraries generated by RepeatModeler, resulted in a decrease of 9%–12% in the previously reported repeat coverages for the original *A. negundo* and *A. saccharum* genomes (McEvoy et al., 2021). The primary reduction was in unknown TEs – those not falling into LTR, DNA, LINE, SINE, or RC classes. LTR coverage decreased slightly (~0.6%), while the resolution on classification improved to the subfamily level. New and original genomes had small differences in counts and coverage of similar elements, while larger trends remained the same.

**FIGURE 5:**
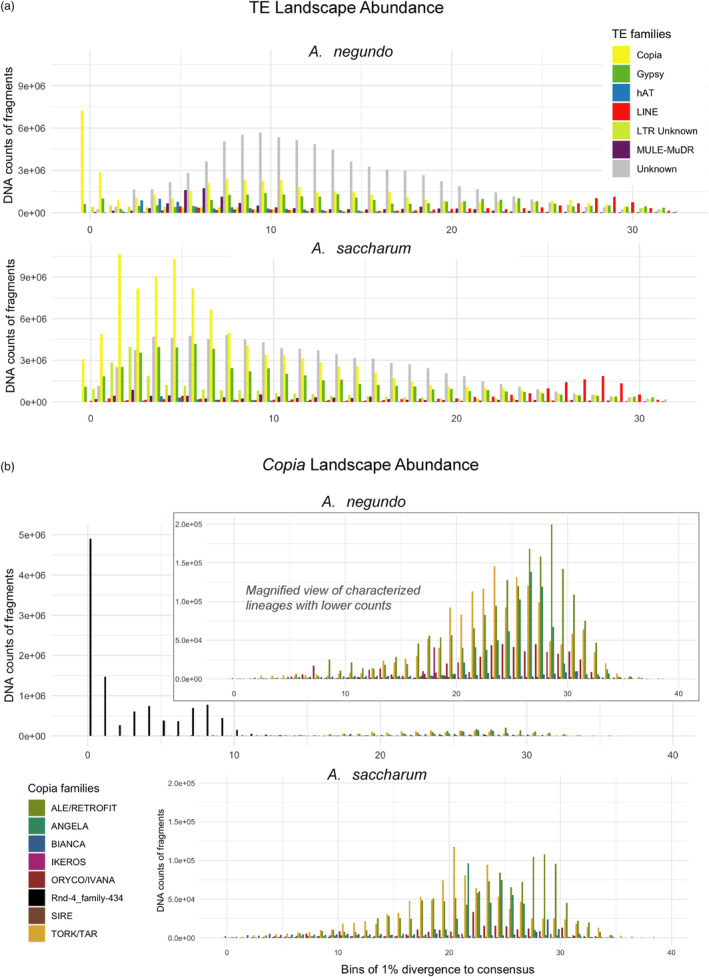
Transposable element abundance for a) predominant repeat families and b) *Copia* subfamilies. The abundance of each repeat class or family, in terms of base pairs, is binned by divergence, with the least divergence representing putative recent activity. In *Acer negundo*, a novel *Copia* repeat identified here as *rnd‐4_family‐434* contributes to the strong recent burst of activity. The other *Copia* subfamilies are provided as an inset for viewability.

The new *A. negundo* genome had 45.2% repeat coverage compared to 45.9% in the original genome. LTRs remained nearly constant at 20% of the genome, with small differences in the family representation. *Copia* had 3369 unique elements covering 11.2% of the new genome compared to 3467 covering 10.6% in the original, followed by *Gypsy* with 2544 unique elements covering 6.4% of the genome and 2437 covering 7.3% of the original (Table 2, File S1). In addition to *Copia* being the largest superfamily, it also appears to have had a burst of recent activity based on the spike in abundance of copies with low sequence divergence (Figure 5a). The greatest subfamily representation is a novel *Copia* identified here as *rnd‐4_family‐434*, which covers 0.026% of the genome compared to the next greatest, *ALE/Retrofit* (0.004%), which has more divergent copies (Figure 5b).

**TABLE 2:** Percent of *Acer negundo* and *Acer saccharum* genomes masked by TE class and *Copia* families.

TE class/family	ACNE (%)	ACSA (%)
LTR	19.74	31.45
Copia	11.23	18.03
ALE/RETROFIT	0.004360	0.002040
ANGELA	0.001740	0.000849
BIANCA	0.000261	0.000223
IKEROS	0.000008	0.000006
ORYCO/IVANA	0.00122	0.00376
Rnd‐4_family‐434	0.026238	N/A
SIRE	0.000115	0.000101
TORK/TAR	0.003390	0.001490
Gypsy	6.40	9.18
SINE	0.01	0
DNA	4.69	2.58
Caulimovirus	0.67	0.45
hAT	1.55	0.97
MULE‐MuDR	2.69	1.35
PIF‐Harbinger	0.19	0.16
RC/Helitron	0.20	0.41
LINE	2.32	2.35
Unknown	18.24	14.13

*A. saccharum* had greater overall total and LTR repeat coverage, and fewer unique *Copia*/*Gypsy* elements that spanned a greater percentage of the genome compared to *A. negundo*. The new *A. saccharum* was estimated to be 50.9% repetitive while the original genome was estimated at 54.5%. LTRs represented 31% and 37% of the new and original genomes, respectively. There were 3092 unique elements of *Copia* with 18.0% overall coverage in the new genome compared to 3015 elements and 22.2% coverage in the original, and only 2294 unique elements of *Gypsy* with 9.2% coverage in the new genome, and 2267 elements with 11.0% coverage in the original. *Copia* subfamilies with lower divergence were even more abundant in these genomes meaning there has likely been somewhat recent activity, but the recent burst noted in *A. negundo* was diminished (Figure 5a,b). *Rnd‐4_family‐434*, the repeat responsible for the recent burst in *A. negundo*, was not found in *A. saccharum*. In examining LTRs across 69 plant genomes, Orozco‐Arias et al. (2023) noted that *Copia* lineages (*Oryco*/*Ivana*, *Retrofit*/*Ale*, *Tork*) were frequently represented with high sequence similarity. The *rnd‐4_family‐434* in *A. negundo* is similar to *Ale/Retrofit* elements.

In most plants, *Gypsy* elements are more abundant and more likely to insert in heterochromatic regions (Vitte et al., 2014), while *Copia* elements are typically more closely associated with genes, more transcriptionally active, and insert in a seemingly random pattern (Galindo‐González et al., 2017; Qiu & Ungerer, 2018). Among the LTRs presented here, both species were dominated by *Copia* elements, followed by *Gypsy*. Other *Acer* recently characterized, including *A. pseudosieboldianum, A. catalpifolium, A. yangbiense*, and *A. truncatum*, demonstrated the same trend, with greater representation of *Copia* elements (Li et al., 2022; Ma et al., 2020; Yang et al., 2019; Yu et al., 2021). Exceptions to this include two maples, *A. palmatum* and the polyploid *A. rubrum*, that host more *Gypsy* elements (Chen et al., 2023; Lu et al., 2022). Notable exceptions to the *Gypsy* dominance have also been observed in *Theobroma cacao*, *Vitis vinifera*, *Musa*, *Rhizophora apiculata*, and *Cucumis sativus* (Argout et al., 2011; Castanera et al., 2019; Moisy et al., 2008; Pratama et al., 2021; Wang et al., 2018). As more genomes become available, more variation in LTR ratios has been noted within and across genera (Zagorski et al., 2020). It is possible that some of this new variation also reflects improvements in long‐read sequencing to resolve and quantify these elements, as seen with *Cucumis melo* (Castanera et al., 2019; Ruggieri et al., 2018). In the *Acers*, despite the fact that the original PacBio reads and the new ONT reads shared similar read N50, the inclusion of the longer ONT reads likely improved the resolution of longer elements. The average length of *Copia* and *Gyspy* elements did in fact increase by several hundred base pairs in *A. negundo*, and while the length of *Copia* elements in *A. saccharum* increased similarly, *Gypsy* elements decreased by 57 bp (File S4).

**FIGURE 6:**
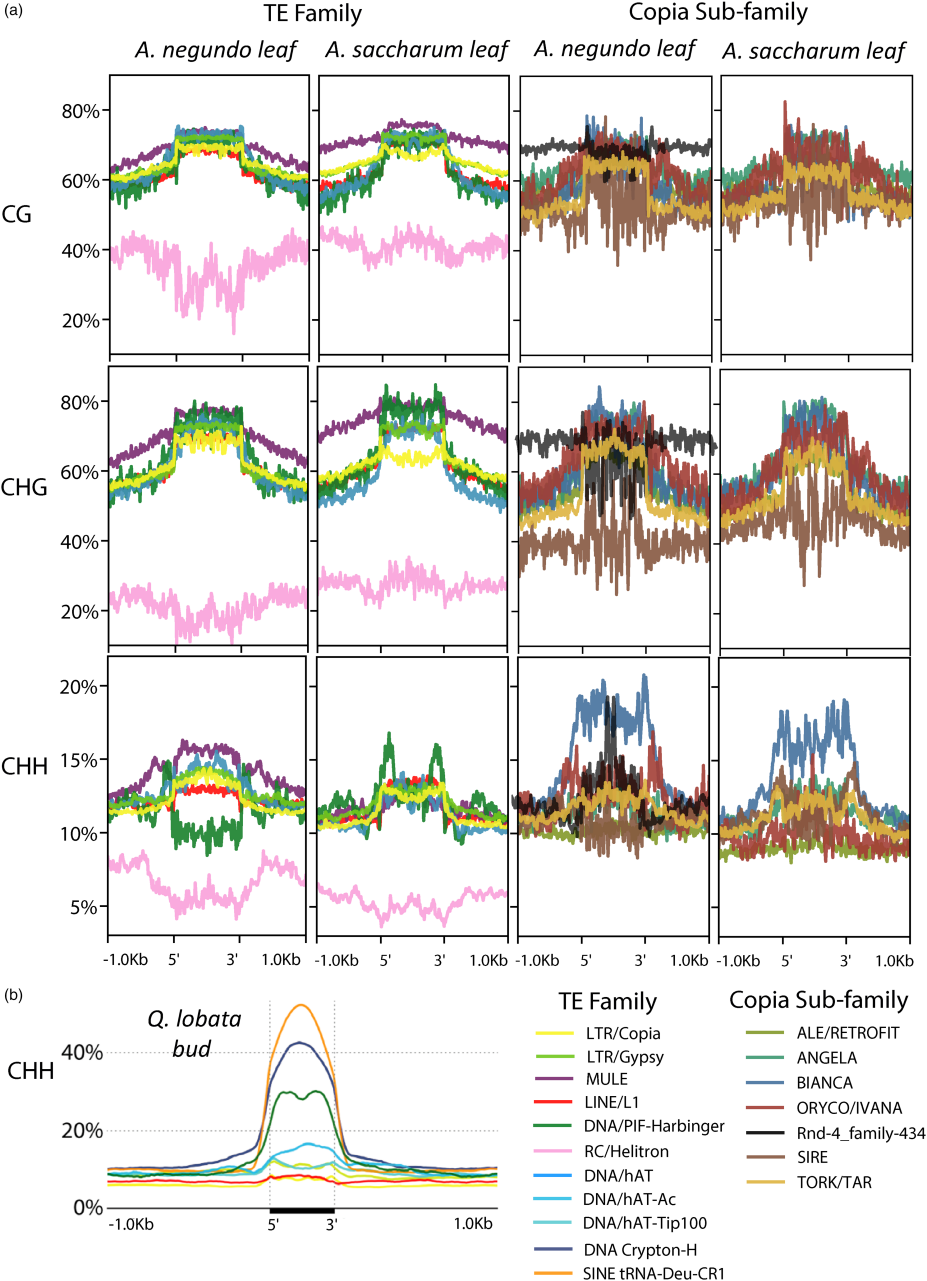
(a) Genome‐wide methylation distribution by sequence context and TE family. (b) Genome‐wide CHH distribution (bud tissue) over TE regions, each line representing one of the largest superfamilies of each class. Reprinted from Sork et al. (2022).

Methylation across repeat superfamilies was plotted along with results from Sork et al. (2022) for comparison (Figure 6a,b), but it should be noted that the *Q. lobata* tissue presented was bud, which contains undifferentiated meristem and had three times the CHH methylation compared to catkin and young leaf tissues. Among the LTRs in *Acer*, *Copias* were more frequent than *Gypsy*, with 4.8% more coverage across the *A. negundo* genome and 8.9% in *A. saccharum*. *Copia* elements were slightly less methylated compared to *Gypsy*, particularly in the CG and CHG contexts, with the largest difference observed in the lower CHG methylation of *Copia* elements in *A. saccharum*. *Copias* were also less methylated in the CHH context of the *Q. lobata* bud tissue (Figure 6b). In both *Acer*, the reduction of CHH methylation in *Copia* is primarily observed in the up and downstream regions. Interestingly, the greatest frequency of *Copia* CHH methylation for each species is highest in *A. negundo* (~14%), followed by *A. saccharum* (~13%), and only ~7% in *Q. lobata*. Greater methylation is commonly observed in *Gypsy* elements and could be attributed to their tendency to reside in heterochromatic regions (Wang et al., 2018).

Methylation across the characterized and dominant *Copia* subfamilies was also examined (Figure 6a). Of the shared *Copia* lineages, copy number ranged from *Tork* which, similar to *A. truncatum* (Ma et al., 2020), had the highest representation in *A. saccharum* (5330) and *A. negundo* (5086), to *Sire* which was most abundant in *A. yangbiense* (6624), but was present in far fewer copies in *A. negundo* (770) and *A. saccharum* (1008). In *A. negundo*, a significant contributor to the recent burst of *Copia* activity and the most abundant, *rnd‐4_family‐434*, had considerably higher CG and CHG methylation in up and downstream regions, and an unusual spike in CHH methylation across the repeat body. In most land plants assessed to date, CHH methylation is observed as a bimodal distribution with most activity in the LTRs themselves (Noshay et al., 2019; Wang & Baulcombe, 2020; Wang et al., 2018). CHH methylation among *Bianca*, represented in far fewer copies, was the highest compared to other *Copia* subfamilies in both species and demonstrated the traditional pattern in *A. saccharum* and one less characteristic (more repeat body methylation) in *A. negundo*. In both *Acer*, *Sire* had less methylation than other *Copia* subfamilies in all sequence contexts except *A. saccharum* CHH. In *A. saccharum*, the CHH methylation frequencies of *Oryco/Ivana* and *Ale/Retrofit* were even lower than *Sire*, and while the copy number of *Oryco/Ivana* was also lower, *Ale/Retrofit* was interesting in its overall prevalence in the genome, with 4035 and 3488 divergent copies for *A. negundo* and *A. saccharum*, respectively. This pattern of high *Ale* sequence diversity and more even distribution through the genome was observed in a population level assessment of *Setaria italica* (Suguiyama et al., 2019). The low CHH methylation observed for Ale/Retrofit elements could be a reflection of sufficient suppression by CG and CHG elements, or it could be ascribed to the inherent structure of these TEs which may influence methylation levels. In *Brachypodium distachyon*, GC content was low in *Sire* and *Bianca*, which was reflected in lower CG and CHG levels, but high in *Ale/Retrofit* with GC content that was decreasing with age due to deamination of cytosines (Stritt et al., 2020).

Studies on CHH methylation in *Arabidopsis* have characterized the unique pathways responsible, and their TE targets (Bouyer et al., 2017). The CMT2 pathway targets the LTRs (primarily *Gypsy* and *Copia*) (Sasaki et al., 2019) and the RdDM pathway targets Class I TEs, specifically RC/*Helitron* and *MULE‐MuDR*. In *Acer*, CHH methylation in DNA transposons was strongly increased (Figure 6a), pointing to a functional role of asymmetric methylation in DNA transposon silencing (Zakrzewski et al., 2017). Among DNA transposons, DNA/*MULE‐MuDR* elements were most highly methylated, except for the CHH context in *A. saccharum*, where it was similar in frequency to other TE families. *MULE‐MuDR* elements contribute to genome crossovers as they are prone to meiotic double‐strand breaks (Underwood & Choi, 2019). The insertion of MuDRs in maize increases meiotic crossover rates and these primarily occur in regions of open chromatin (Liu et al., 2009). The loss of CG and non‐CG methylation over MuDr elements in *Arabidopsis* mutants led to an increase in crossovers that were enriched in proximity to immunity genes of large and diverse families with extensive variation (Choi et al., 2018). DNA/*PiF‐Harbinger* elements were also highly methylated across the gene body with the exception of CHH methylation in *A. negundo*. CHH methylation of *PiF‐Harbinger* has a bimodal distribution, with peaks flanking the element in both *Acer*s, similar to *Q. lobata* (Figure 6b), but the overall frequency of CHH methylation is much higher in oak (~30%) which may be a tissue‐specific pattern. *PiF‐Harbinger* elements only cover a very small portion of the *Acer* genomes at ~0.18%, but they are widespread throughout plant lineages, at times in great abundance (~1%) relative to most DNA transposons as observed in *Arabidopsis thaliana*, *Oryza sativa*, and *Brassica oleracea* (Zhang et al., 2004). They have been associated with important adaptive roles, including intronic *Harbinger* elements that contain MYB elements that regulate stress response to light in *Solanum lycopersicum* (Deneweth et al., 2022), and a *Harbinger*‐derived gene that coordinates histone modifications that alter panicle number and grain size in *O. sativa* (Mao et al., 2022).

Among non‐LTR retroelements, *SINE*s were heavily methylated (>40% for CHH) in *Quercus* bud tissue (Figure 6b) but not frequently identified in the *Acer* for comparison (File S1). *SINEs* are derived from tRNA and demonstrate low sequence similarity which may be responsible for their poor annotation (Wenke et al., 2011). Recent analysis across eight grass genomes examined patterns in mCHH islands and found significant enrichment of these islands in DNA transposons (DNA/*MULE‐MuDR, PiF‐Harbinger)* as well as non‐LTR retroelements (SINEs) (Martin et al., 2021).

**FIGURE 7:**
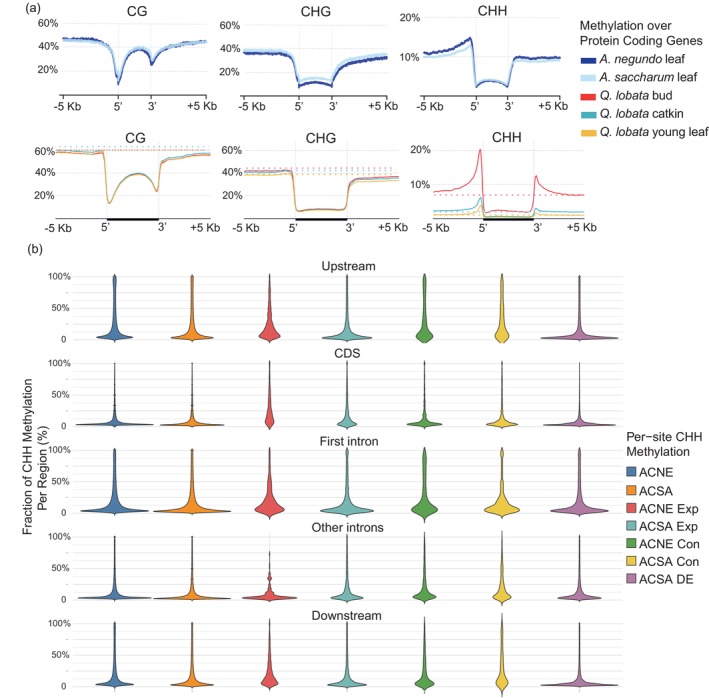
(a) Methylation frequency distribution across protein coding regions, shown by genome, tissue type, and sequence context, (b) Methylation frequencies in different genic regions (1 Kb upstream of TSS, CDS, first intron, other introns, and, 1 Kb downstream of the TES). ACNE/ACSA: genome‐wide means, Con: rapidly contracting genes (ACNE: 52, ACSA: 18), Exp: rapidly expanding genes (ACNE: 26, ACSA: 99), DE: differentially expressed genes. Exp and Con genes are from McEvoy et al. (2021), as well as DE, where genes were differentially expressed in response to calcium and aluminum treatment. Of the original 245 DE genes identified from stem tissue samples, 240 are presented here with methylation calculated from leaf tissue.

Methylation frequencies across gene regions in *Acer* were somewhat different than that observed in *Q. lobata*, but there is a spectrum of variation observed across angiosperms, as seen in Niederhuth et al. (2016) (Figures 4a and 7a,b). The two *Acer* species were very similar, but *A. negundo*, which has the smaller genome, had a slightly higher frequency of CG methylation. The higher frequencies across genes in the original genomes compared to the new genomes may be due to differences in average gene length, as seen in *Q. lobata* (Figures 7a and S4). Both *Acer* species also shared the same sharp increase in CHH just upstream of the transcription start site, which was greater in *A. negundo*.

CHH levels were highest in the 5 Kb region upstream of the transcription start site (TSS) (Figure 7a,b). The next largest fraction was in the first intron, where both new *Acer* genomes had a significant drop in the number of methylated sites, especially for those at 90% methylation (Figure 7b). This pattern was seen in other introns, but at lower levels, while the levels in the 5 Kb region downstream of the transcription end site (TES) were consistent across frequencies. In certain plant genes—often highly expressed genes—the first intron contains regulatory elements, though it is observed much less frequently than upstream promoters (Rose, 2018).

### 3.6 Methylome and gene regulation

**FIGURE 8:**
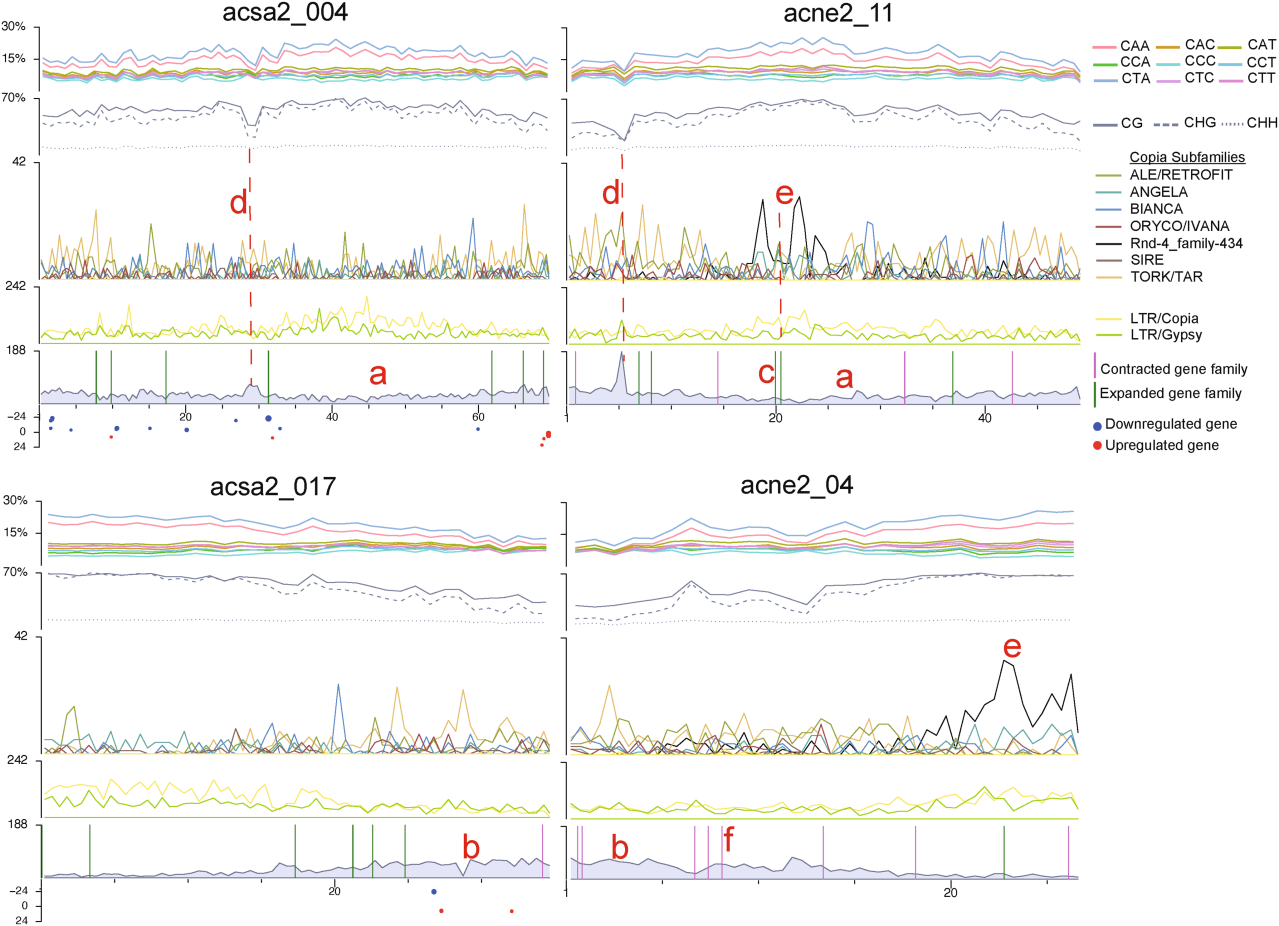
Distribution of gene density, repeat families, methylation by context, CHH methylation subcontexts, gene family dynamics, and gene expression results for select chromosomes (all chromosomes available in Figure S5). Gene expression results are from aluminum and calcium treatments at Hubbard Brook Experimental Forest as detailed in McEvoy et al. (2021). X‐axis indicates log2 fold change, while dot size represents the *p*‐adjusted value. Letters mark examples as follows: (a) even distribution of gene density, (b) concentrated gene density, (c) *Acer negundo* chromosome with the most genes from expanded families (d) drops in methylation over spikes in gene density, (e) peaks of *Copia* rnd‐r_family‐434, (f) *A. negundo* chromosome with genes from contracted families.

By combining distributions of methylation, select TEs, and gene density, trends amongst the elements can be observed. In Figure 8, each row contains chromosomes that are largely syntenic between *A. negundo* (ACNE) and *A. saccharum* (ACSA), as seen in Figure 2b, with all chromosomes from each genome available in Figure S5 for more detailed viewing. Regions of low gene density are co‐located with peaks of LTRs and methylation in all contexts, including CHH. It is likely these contain centromeres and pericentromeric regions. Included in the gene density track (Figure 8) are genes from families previously identified as significantly expanding (26 in *A. negundo*, 99 in *A. saccharum*) or significantly contracting (52 in *A. negundo*, 18 in *A. saccharum*) when evaluated in terms of gene family evolution across 22 land plant species (McEvoy et al., 2021). Genes from rapidly evolving families are found in every chromosome except acsa2_011, though some contain many more than others (Figure S5).

The particular subset of chromosomes shown in Figure 8 provides a few different examples of distribution patterns of methylation and TEs and rapid gene family evolution in chromosomes. The top set of syntenic chromosomes, acsa2_004 (*A. saccharum*) and acne2_11 (*A. negundo*), shows a more even distribution of gene density rather than a concentration of genes in the chromosome arms. The *A. negundo* chromosome (acne2_11) had the most genes from expanded families in that genome, but also contains some genes from contracted families. There is a notable drop in methylation over a narrow peak of gene density in both species that is greater in *A. negundo*. *A. negundo* also has spikes of high frequency *rnd‐4_family‐434* that correspond to the greatest methylation frequencies and lowest gene densities. *Rnd‐4_family‐434*, the Copia subfamily with the burst of recent activity, does not appear to be proximal to rapidly evolving genes (Figure S5), but there are instances where the two overlap, as shown in acne2_11, where two genes from rapidly expanding families fall between the two spikes of *rnd‐4_family‐434*.

The second set of chromosomes, acsa2_017 and acne2_04, are also syntenic, but inverted, and gene density is concentrated in one arm of each species. In this distribution, *rnd‐4_family‐434* is concentrated at the opposite end of the *A. negundo* chromosome where gene density is the lowest and methylation the greatest. This distribution pattern, weighted towards one chromosome arm, tends to be more common genome‐wide in both species (Figure S5). The *A. negundo* chromosome has several genes from contracted families and one from expanded, while *A. saccharum* is the inverse, which fits the predominant direction of rapidly evolving gene families for each species. In the previous *Acer* study, *A. negundo* was shown to have more families associated with rapid contraction and very few expanding, and here, expanded gene families show an increase in CHH methylation in the gene body (coding regions) relative to other categories of genes in both species (Figure 7b), but it is greater in *A. negundo*. *A. saccharum* primarily had expanding families, and here, genes from contracted families have distinct increases in CHH methylation upstream, downstream, and in the intronic space, regardless of position (Figure 7b).

Several studies have examined the relationship between methylation changes, metal toxicity, and the associated nutrient stress, in both model and non‐model plant systems. One such study examined the aquatic plant *Hydrilla verticillata* and noted demethylation in response to copper, as well as blocked ROS interactions that could cause remethylation (Shi et al., 2017). In *Hibiscus cannabinus*, increased methylation was associated with increasing chromium levels (Ding et al., 2016). In *Arabidopsis halleri*, cadmium treatments increased CG methylation of genes responsible for symmetric methylation (Galati et al., 2021). In *O. sativa*, a model for nutrient stress, the expression of Heavy Metal Transporting ATPases (HMAs) was modified and retained over generations and modulated by the methylation of specific TEs (Cong et al., 2019). Gallo‐Franco et al. (2022) found that aluminum tolerant and susceptible varieties of *O. sativa* and *Oryza glumaepatula* each had unique differentially methylated regions, and each species was associated with different TEs potentially affecting regulation of these regions. While relationships between environmental stress, methylation, and increased proximal TE activity have been observed in numerous studies, this regulation is complex and potentially involves specific methylation mechanisms, such as the *Copia ONSEN* in both *Arabidopsis* and *Oryza*. This TE inserts near histone variant H2A.Z, the deposition of which can be regulated by environment (Baduel & Quadrana, 2021). While our study does not lend itself to proper examination of species‐specific patterns of methylation that can be correlated to gene expression changes, the potential for gene regulation among candidate genes previously identified in RNA‐Seq experiments in *A. saccharum* could be examined.

Gene expression results focused on candidates associated with nutrient stress (or heavy metal toxicity) were mapped to the new *A. saccharum* genome (240 in total; Figure 8). A total of 115 genes were downregulated in trees grown in aluminum‐amended plots while 130 genes were up‐regulated (in both cases, compared with trees grown in calcium‐amended or control plots). These genes are distributed across all chromosomes at different densities and it is difficult to detect trends from this perspective. To further examine methylation of the 240 differentially expressed genes, they were compared with the patterns observed for the mean of all genes in Figure 7b and File S4. The subset of differentially expressed genes appeared enriched for CG and CHG methylation in the upstream regions when compared with the full gene space. The most noticeable difference, however, was the higher peak of CHH methylation frequency immediately preceding the transcription start site in the promoter region of differentially expressed genes (Figures S4 and 7b) which has been observed in grasses and other angiosperms (Martin et al., 2021). Values here peak at around 16% compared to 12.5% for the whole‐genome mean. This value is comparable to the CHH methylation observed in *Q. lobata* bud tissue relative to catkins or young leaves, which have a greater portion of meristematic tissue, as mentioned in Sork et al. (2022), and are presumably undergoing rapid development. Increased methylation could be a sign of gene networks requiring more flexibility in expression to meet the challenges of development or biotic and abiotic stress (Lang et al., 2017).

## 4 CONCLUSION

Plants are dependent on methylation to regulate both short and long‐term adaptive strategies (Sun et al., 2022). Best practices to detect this methylation leading to an understanding of integrated epigenomic data are important for a full understanding of the genomic mechanics in effect. To extend previous work on DNA methylation detection and integration, this study developed two improved reference genomes for two new accessions of *A. saccharum* and *A. negundo*. These hybrid assemblies benefitted from the inclusion of deep ONT coverage to both resolve long repeat elements and detect methylation. Methylation calling was conducted with ONT sequencing data and a custom pipeline. This pipeline, which leverages super accuracy base calling and detection of false positives, has broader application for other non‐model plant genomes. Methylation frequencies and distributions were compared with other recent plant methylomes and revealed differences among the species where *A. saccharum* and *A. negundo* have a lower correlation of methylation and gene content than many angiosperms and fall in the middle of the spectrum of gene‐dense arms with low methylation of *P. trichocarpa* and the generalized distribution of methylation in *Q. lobata* and *Poaceae*. In both *Acer* species, the repeat landscape was dominated by LTR/*Copia* elements. A recent burst of a novel *Copia* in *A. negundo* showed higher than average CG and CHG methylation up and downstream and an unusual pattern of strong CHH methylation in the repeat body. Gene family dynamics integrated with methylation data noted that individual genes in contracted families were associated with strong CHH methylation upstream, downstream, and in intronic regions. Expanded gene families had more CHH methylation in coding regions than contracted families. Among candidate genes previously associated with nutrient stress, patterns of methylation were variable, but had increased upstream methylation observed in all three contexts. Further investigations require parallel expression and tissue‐specific studies, as well as pan‐genome (population scale) analysis to understand how the methylome is contributing to genome evolution.

## AUTHOR CONTRIBUTIONS

Susan L. McEvoy and Jill L. Wegrzyn designed research and wrote the paper. Susan L. McEvoy performed computational analyses. Nicole Pauloski performed laboratory work and generated sequencing data. Patrick G. S. Grady performed re‐basecalling of ONT data. Rachel J. O'Neill provided compute and personnel resources.

## CONFLICT OF INTEREST STATEMENT

The authors declare no conflict of interest.

## FUNDING INFORMATION

Funding was provided by the Botanical Society of America, Bill Dahl Graduate Student Research Award and The Ronald Bamford Endowment Fund for Botany Research to the Department of Ecology and Evolutionary Biology. PGSG was supported by National Institutes of Health R01GM123312–02 to R. O'Neill.

## Supporting information


**Figure S1.** Distribution of CHH methylation by subcontext across all chromosomes (1 Mbp window every 1 Mbp) in new and original *Acer negundo* (acne) and *Acer saccharum* (acsa) genomes.

**Figure S2.** Distribution of CHG methylation by subcontext across all chromosomes (1 Mbp window every 1 Mbp) in new and original *Acer negundo* (acne) and *Acer saccharum* (acsa) genomes.

**Figure S3.** Gene densities (red) with CG (blue), CHG (green), and CHH (maroon) contexts in 100 Kb windows across new and original *Acer negundo* (acne) and *Acer saccharum* (acsa) genomes.

**Figure S4.** (a) Methylation frequency distribution across protein coding regions, 5′ to 3′, shown by assembly and sequence context for new *Acer negundo* (acne2) and *Acer saccharum* (acsa2) genomes. Top row includes results from whole‐genome mean, second row is genes from rapidly expanding gene families, third row is genes from rapidly contracting families. Bottom row shows methylation frequency distribution across 240 (of the original 245) genes differentially expressed in response to calcium and aluminum treatments in stem as seen in McEvoy et al. (2021).

**Figure S5.** Chromosome‐level distribution of gene density, LTR repeat families and Copia subfamilies, methylation by context, CHH methylation subcontexts, gene family dynamics, and gene expression results for select chromosomes. New *Acer negundo* (acne2) and *Acer saccharum* (acsa2) are shown. Genes from expanded gene families are shown as lime green vertical bars, and those from contracted, as purple bars. *Acer saccharum* gene expression results (red and blue dots) are from aluminum and calcium treatments at Hubbard Brook Experimental Forest as detailed in McEvoy et al. (2021). X‐axis indicates log2 fold change, while dot size represents the *p*‐adjusted value.

**FILE S1**. Assembly statistics, annotation statistics, summaries of repeat coverage by class and family, tombo resquiggle summaries https://gitlab.com/PlantGenomicsLab/acermethylation/‐/blob/main/manuscript/supplemental/FileS1‐summaries.xlsx.

## Data Availability

*Acer saccharum* reads are deposited in the SRR15192908‐14 and the genome in WGS JAUUCP000000000 (BioProject PRJNA748028). *Acer negundo* reads are deposited in SRR17273852‐58 and the genome in WGS JAUKTQ000000000 (BioProject PRJNA750066). Previously published Acer genomes are available in the same respective BioProject IDs. All assemblies and annotations will also be made available in TreeGenes (https://treegenesdb.org/). Scripts are available at doi: 10.5281/zenodo.10659005.
