# Supplementary figures and images for: Profiling genome‐wide methylation in two maples: Fine‐scale approaches to detection with nanopore technology
Source: Evol Appl. 2024 Apr 17;17(4):e13669. doi: 10.1111/eva.13669 (PMC11022628; doi:10.1111/eva.13669)

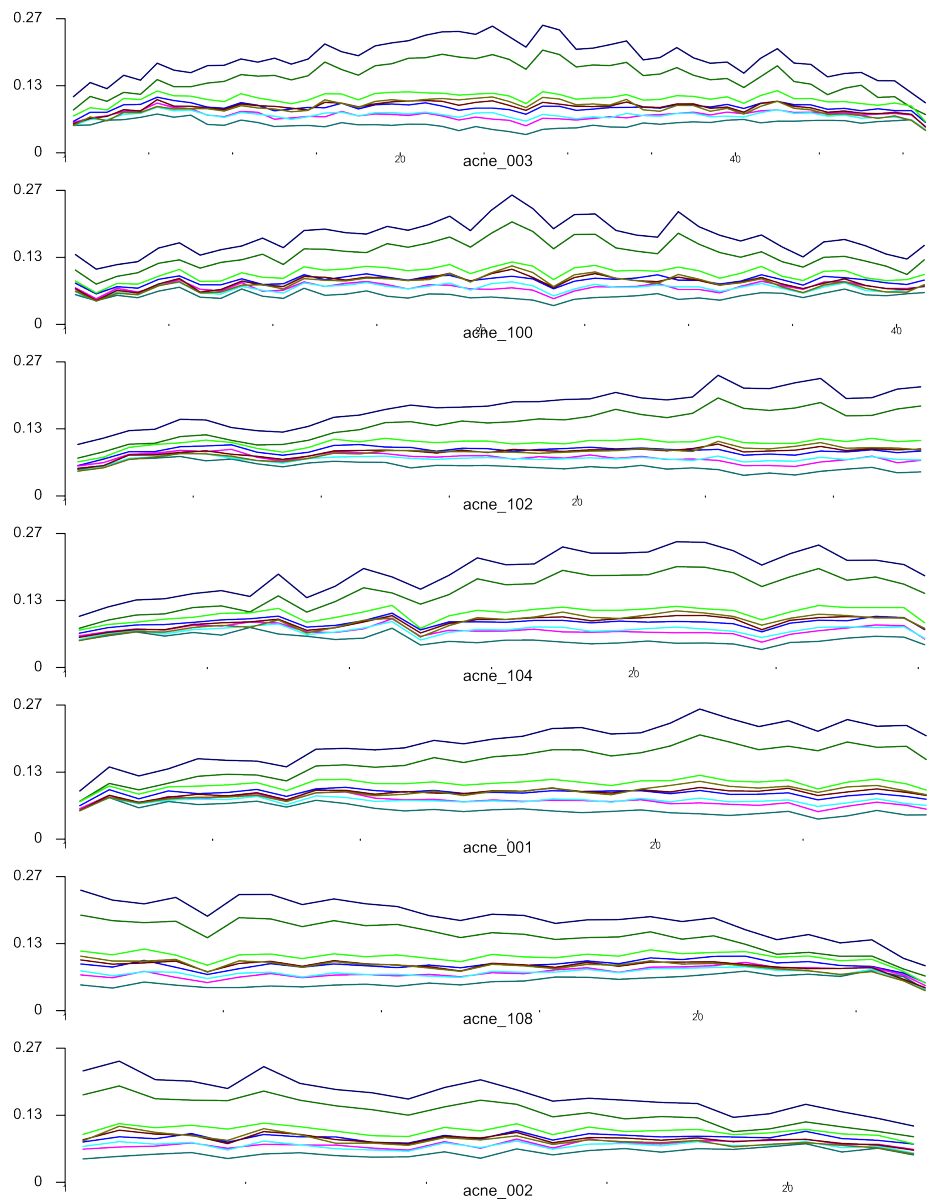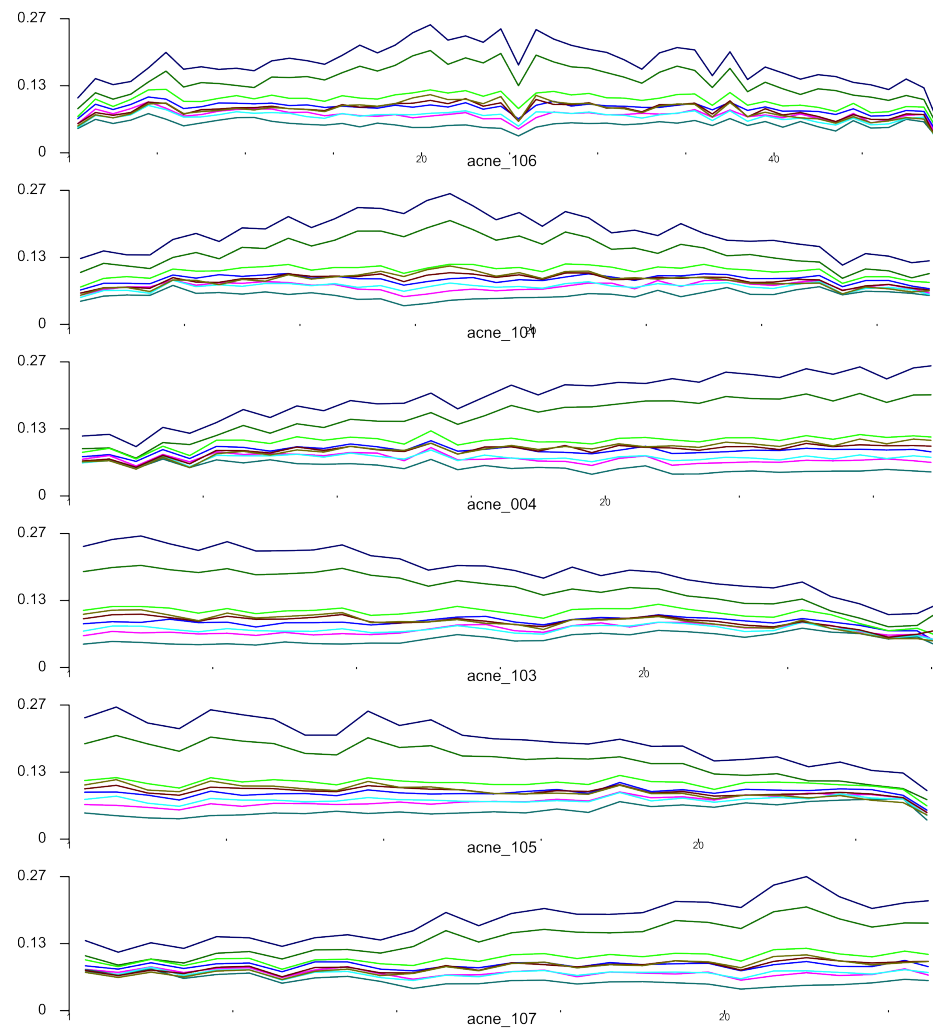

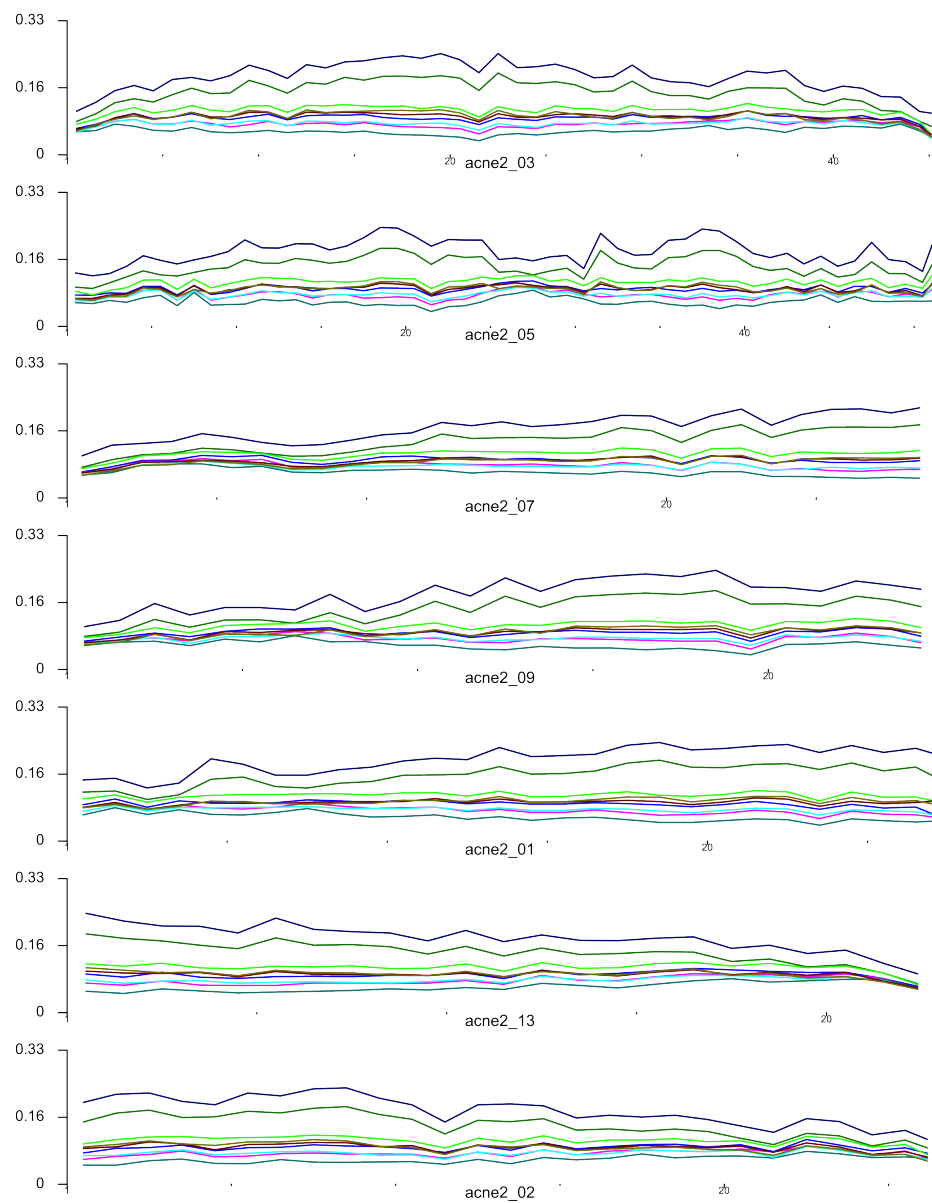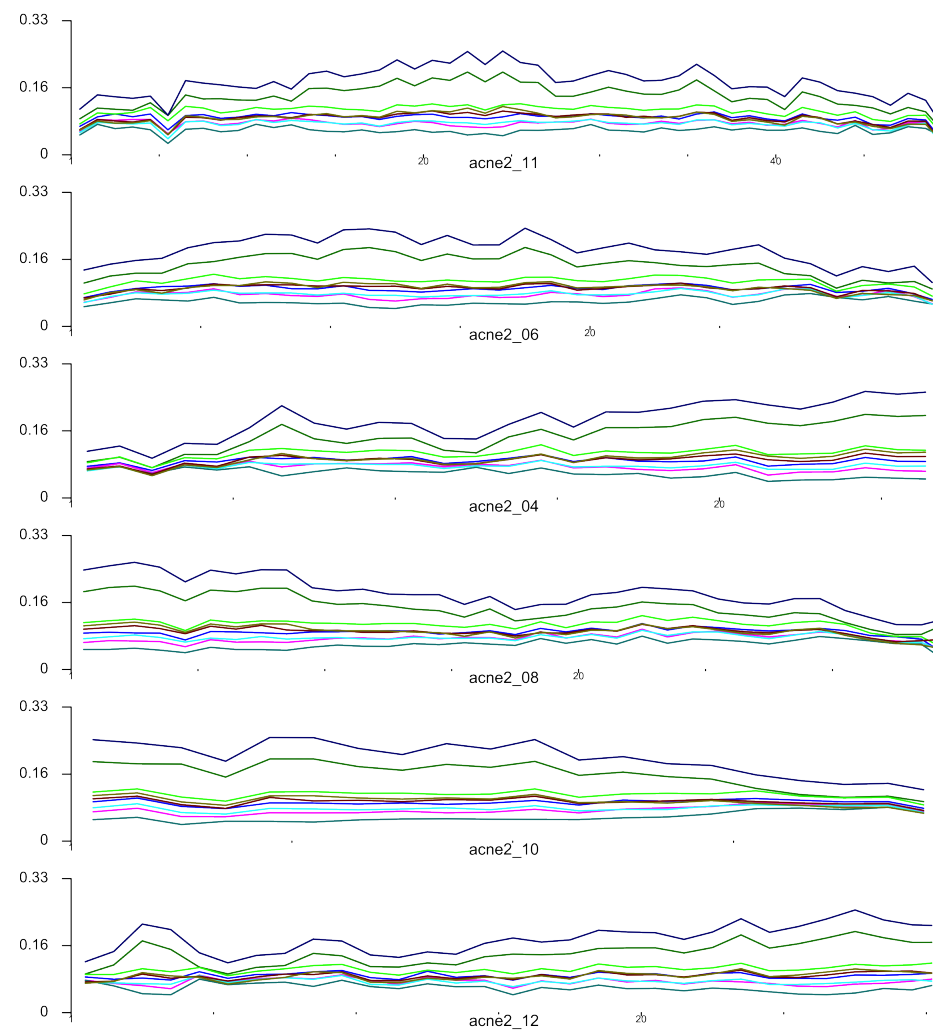

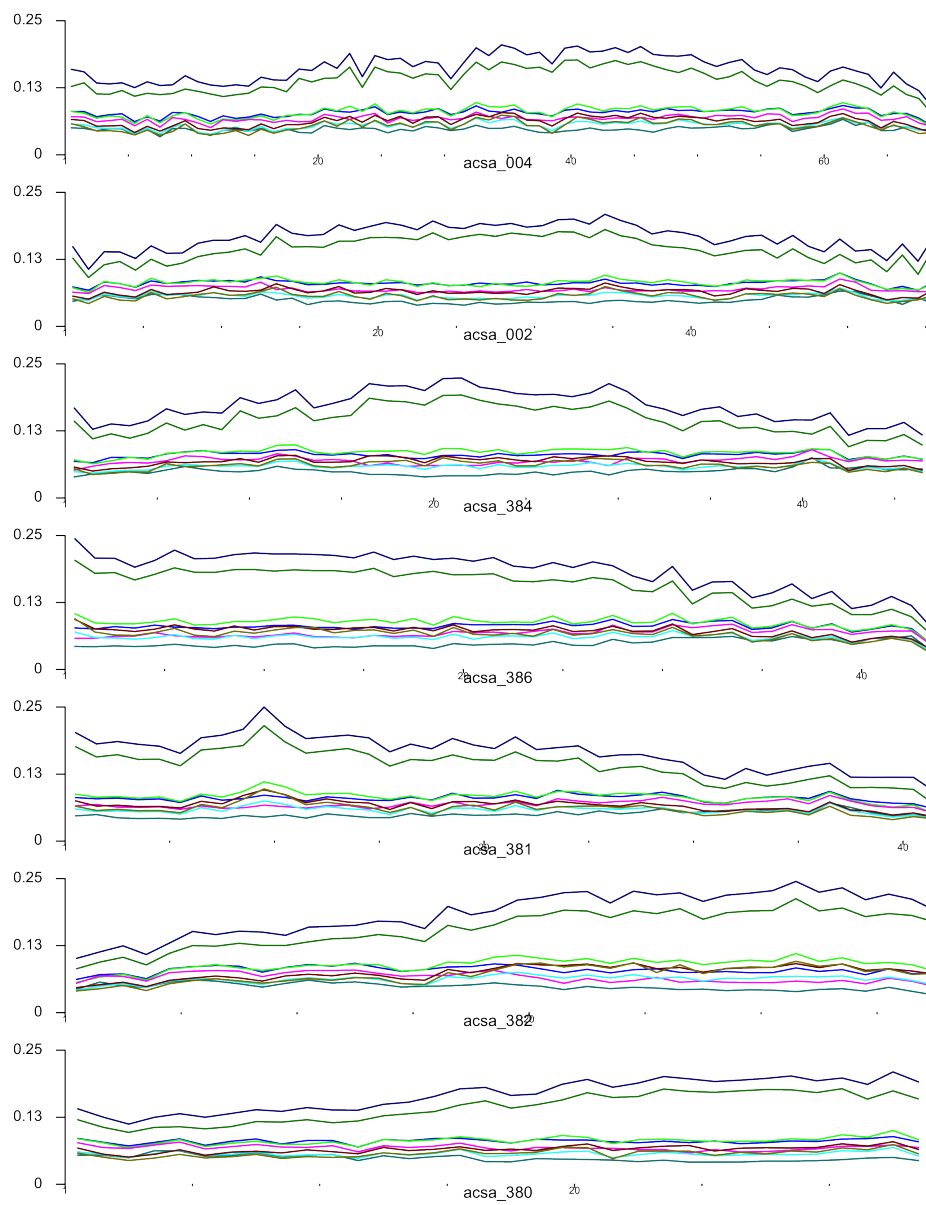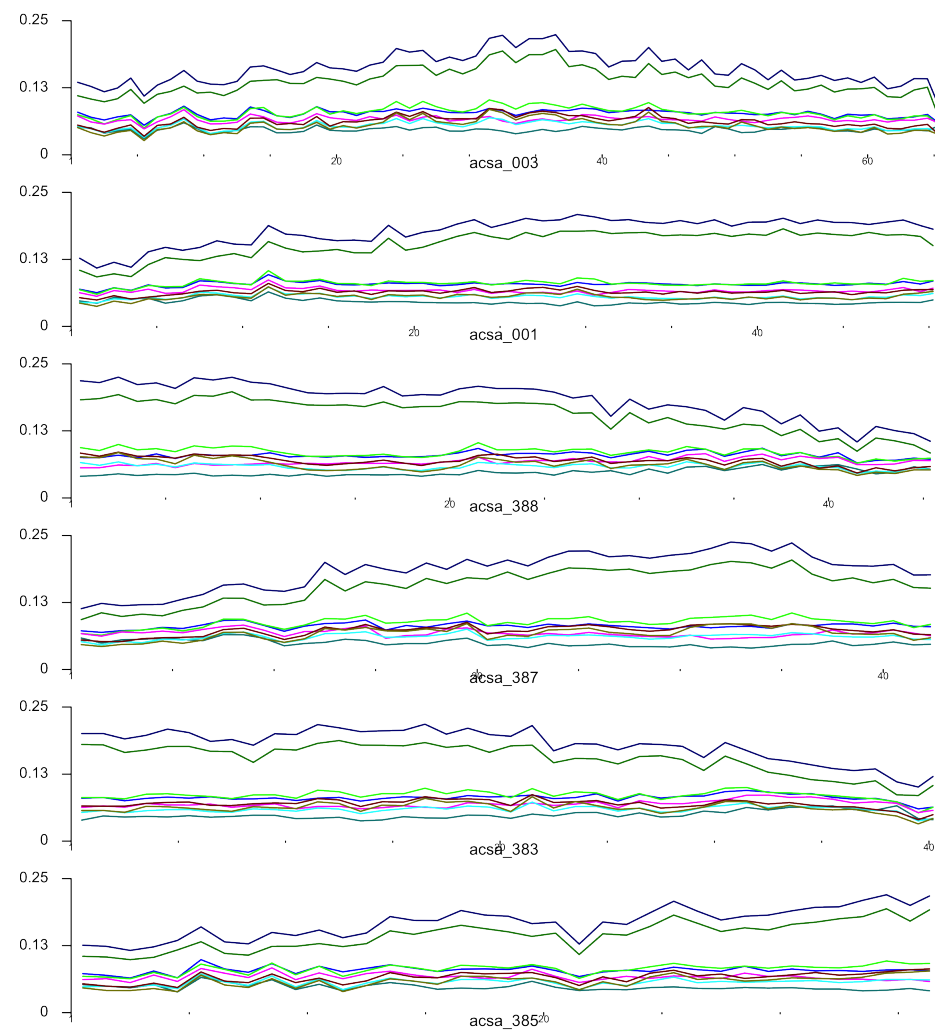

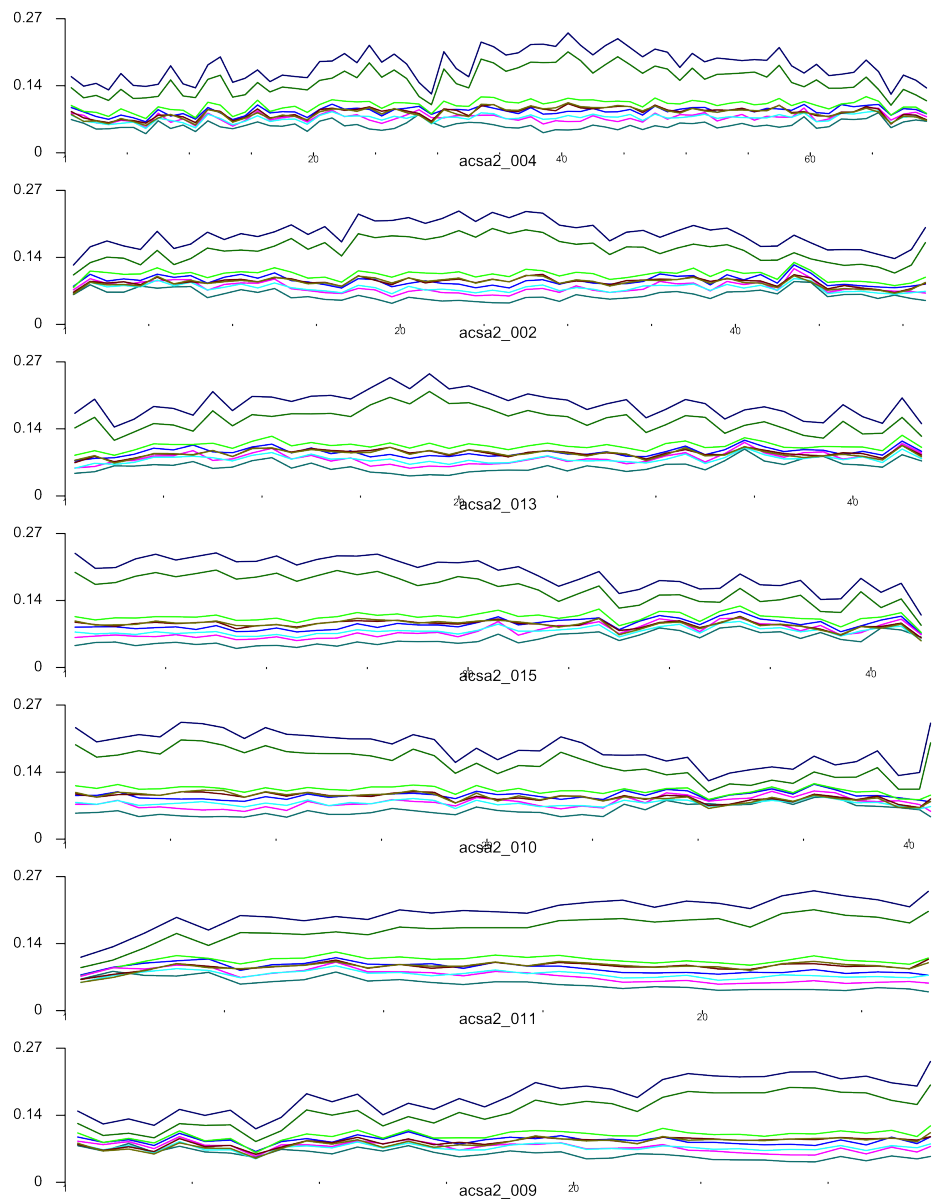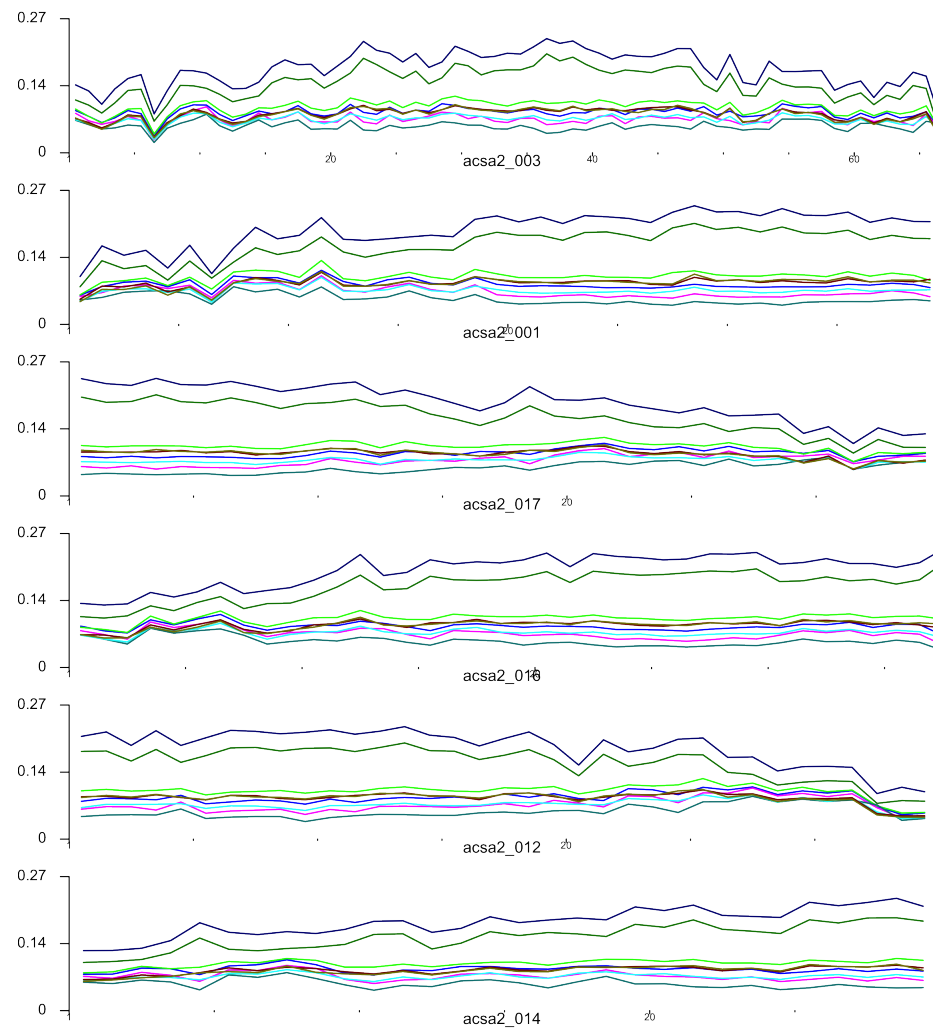

Supplement: Supplementary file 1 — Figure S1. Distribution of CHH methylation by subcontext across all chromosomes (1 Mbp window every 1 Mbp) in new and original Acer negundo (acne) and Acer saccharum (acsa) genomes. [file EVA-17-e13669-s002.pdf]

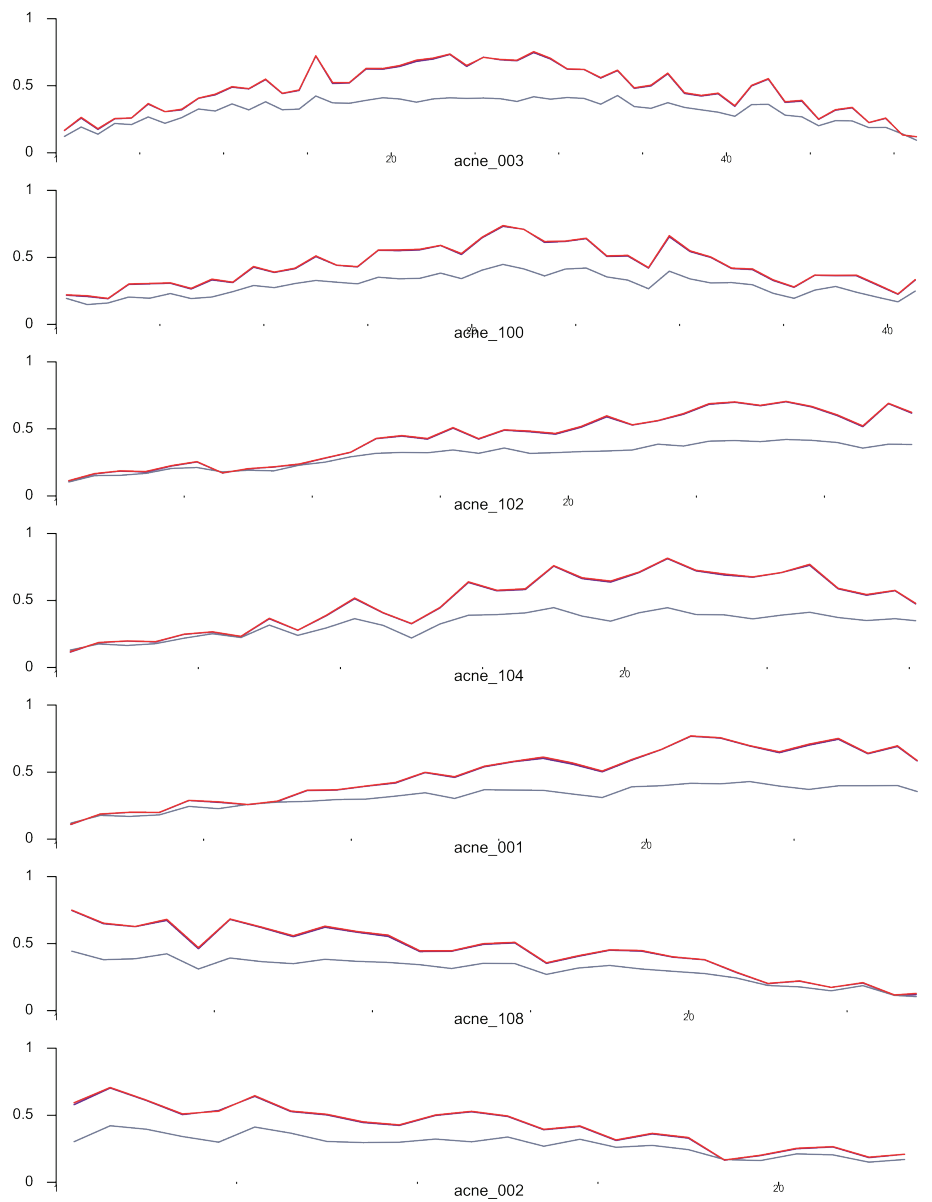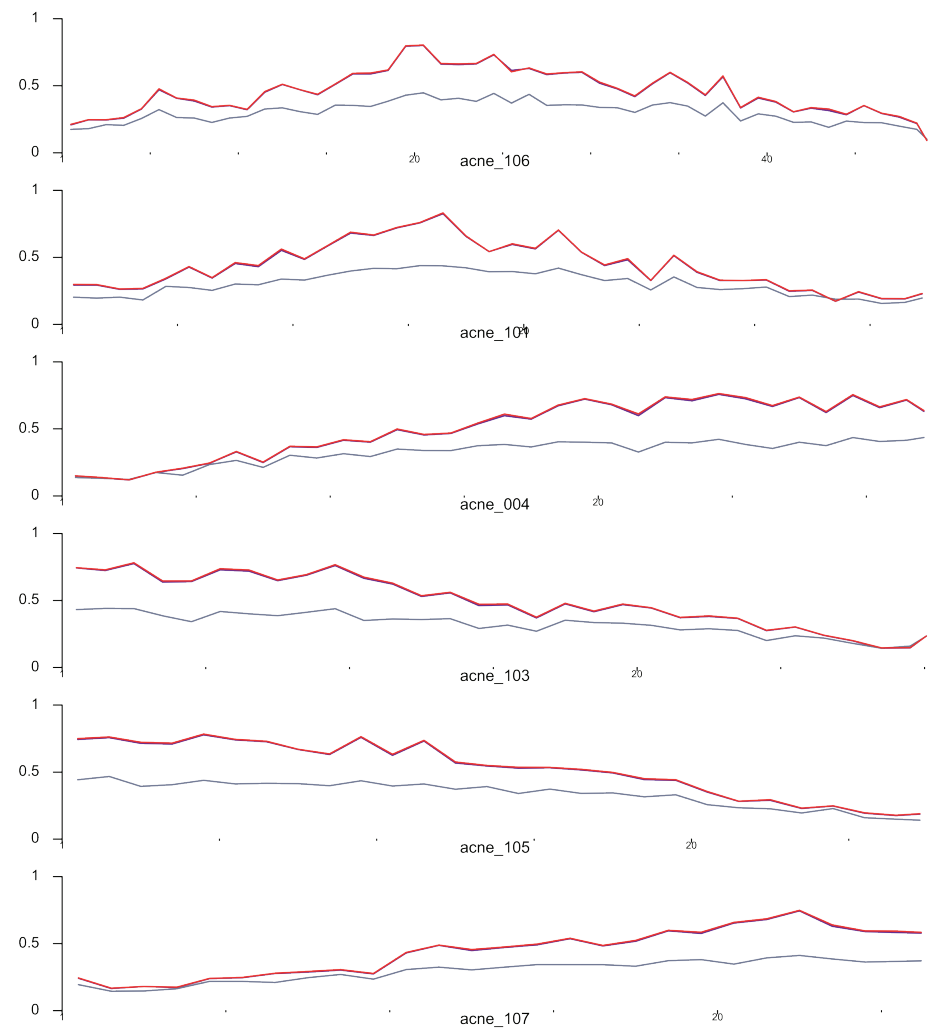

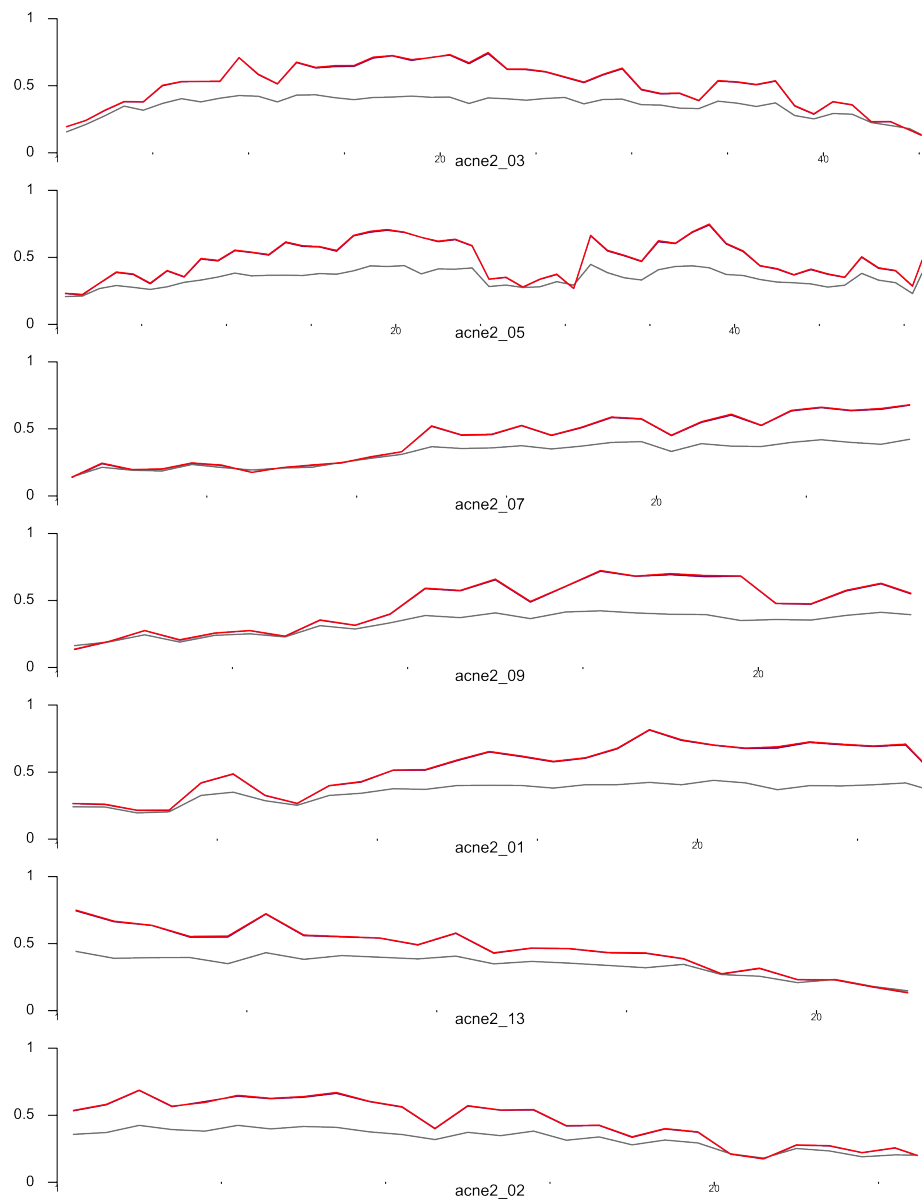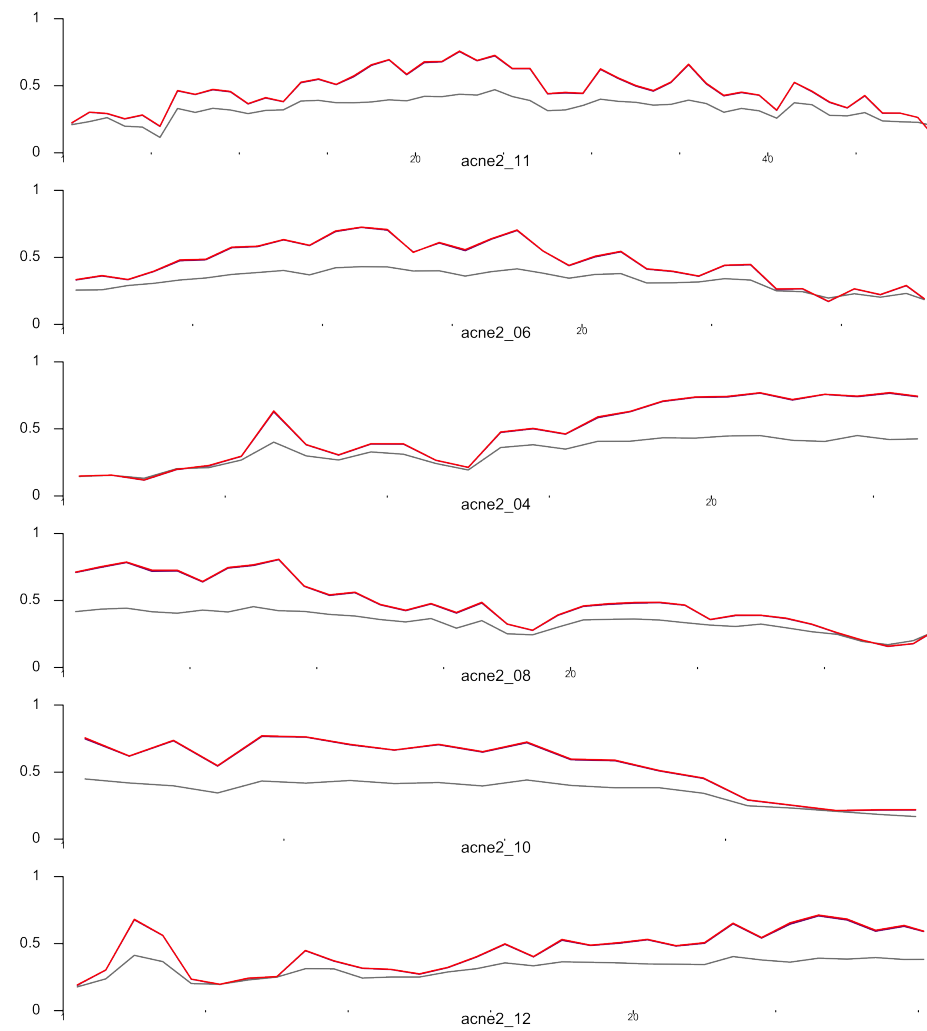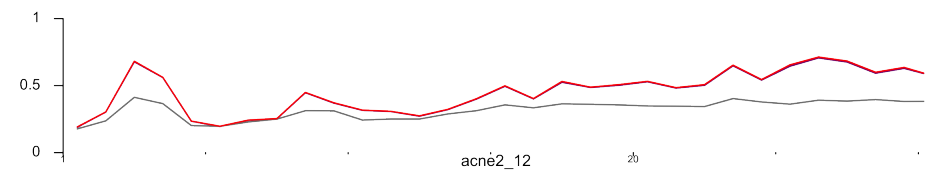

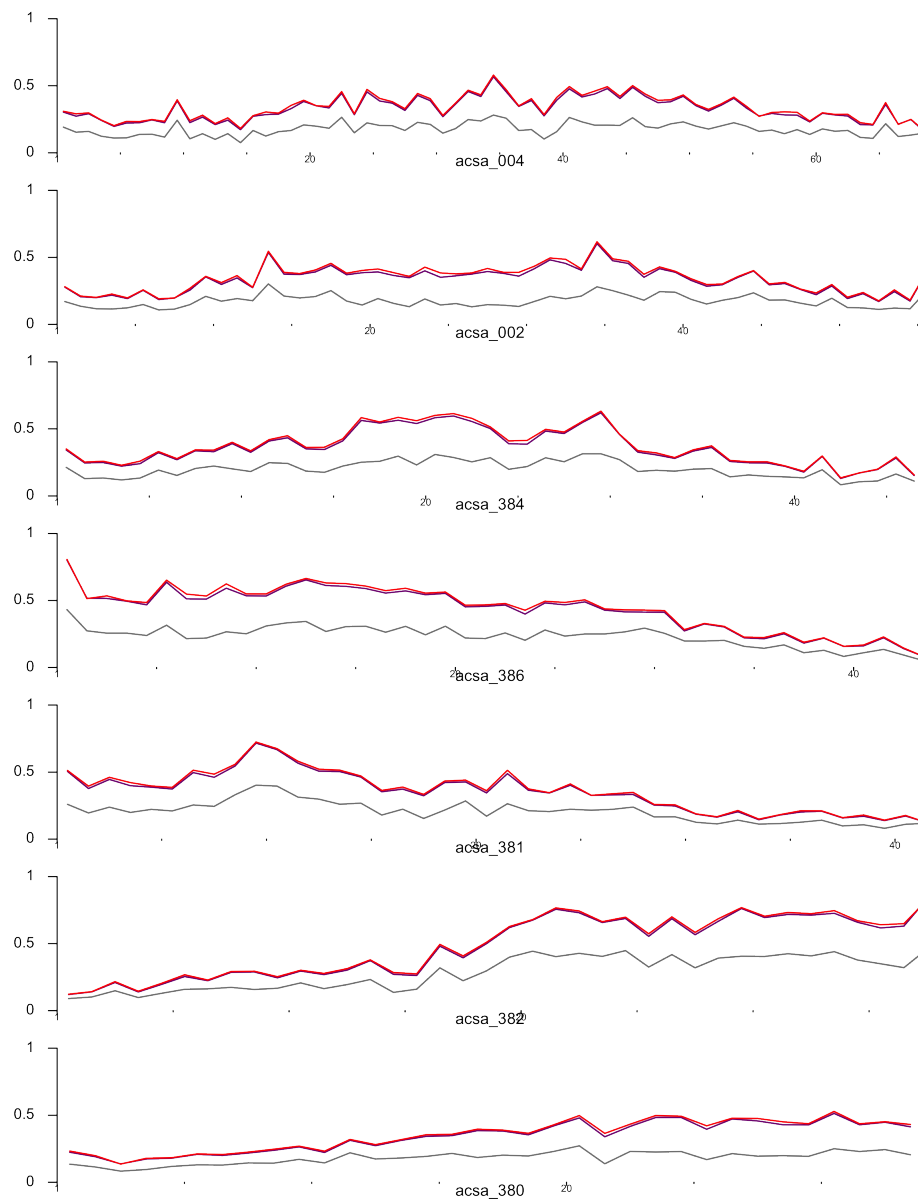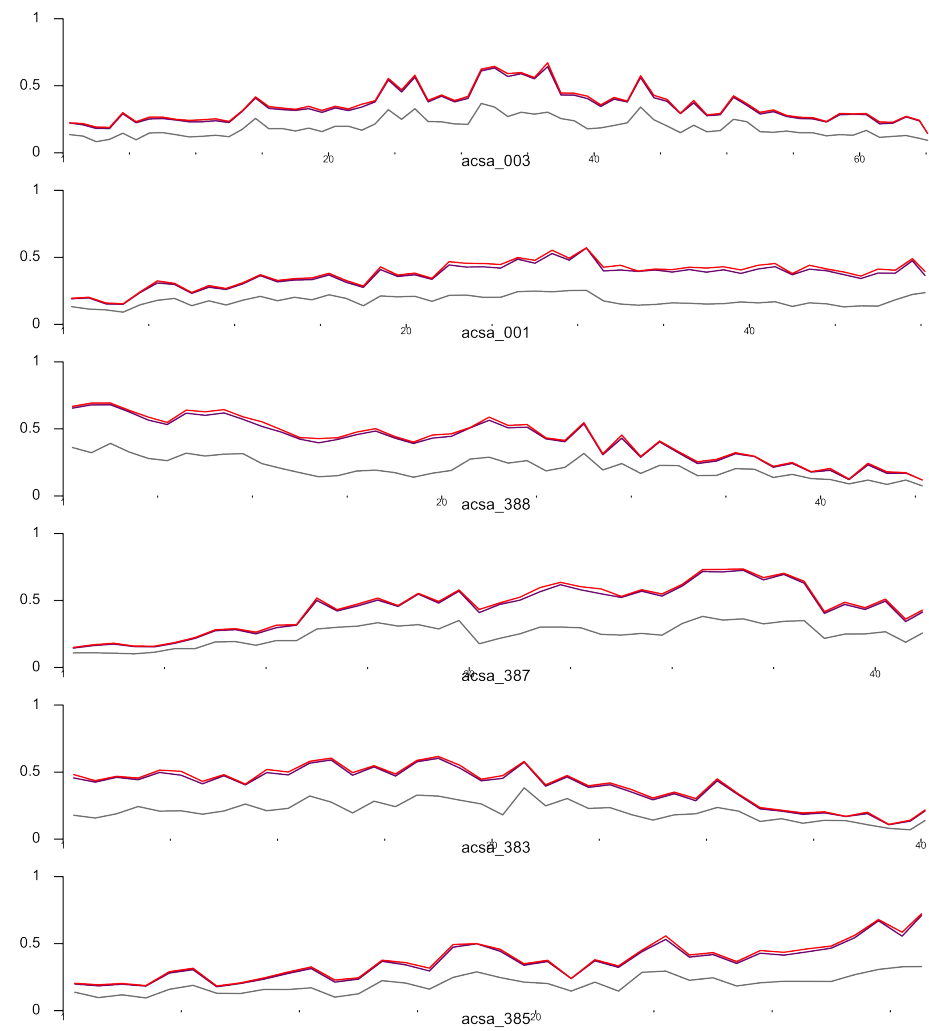

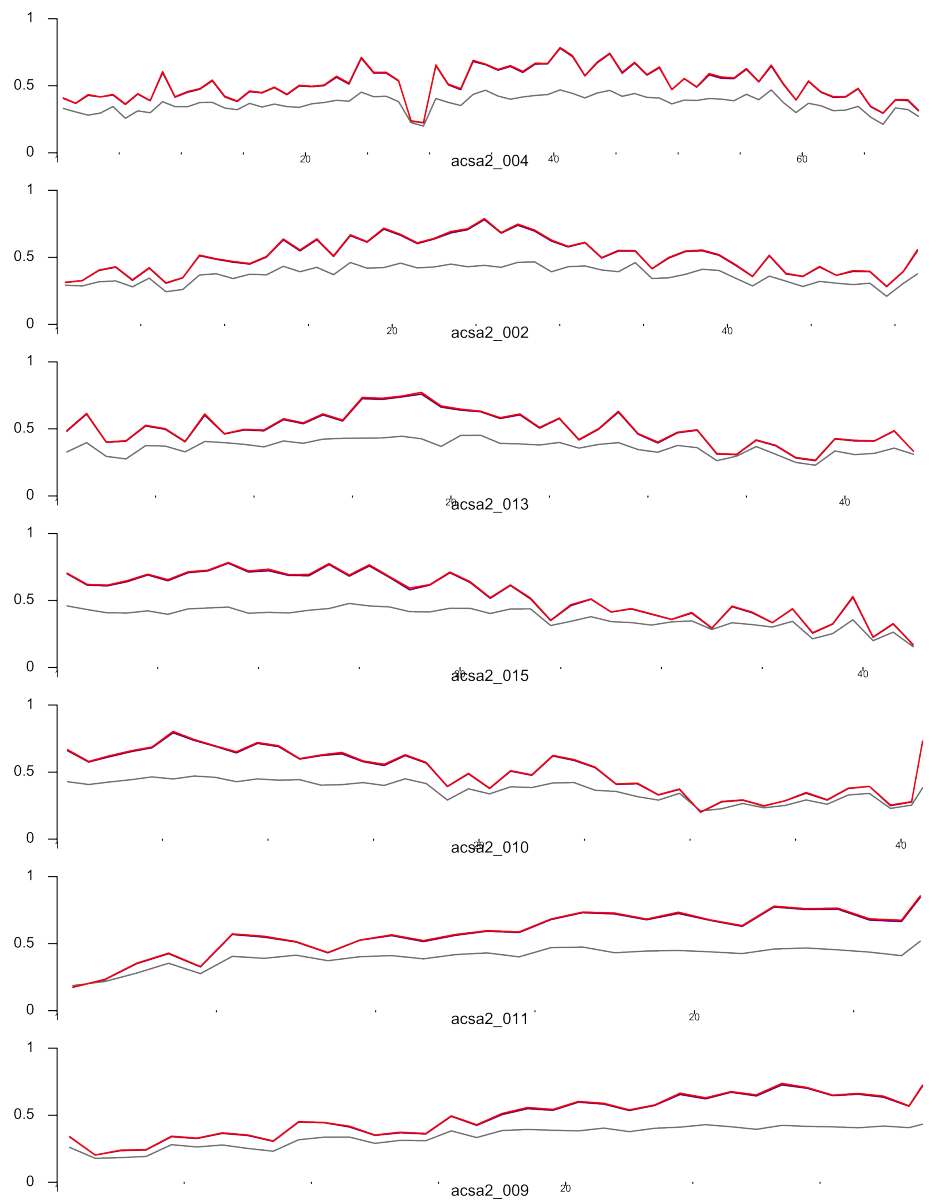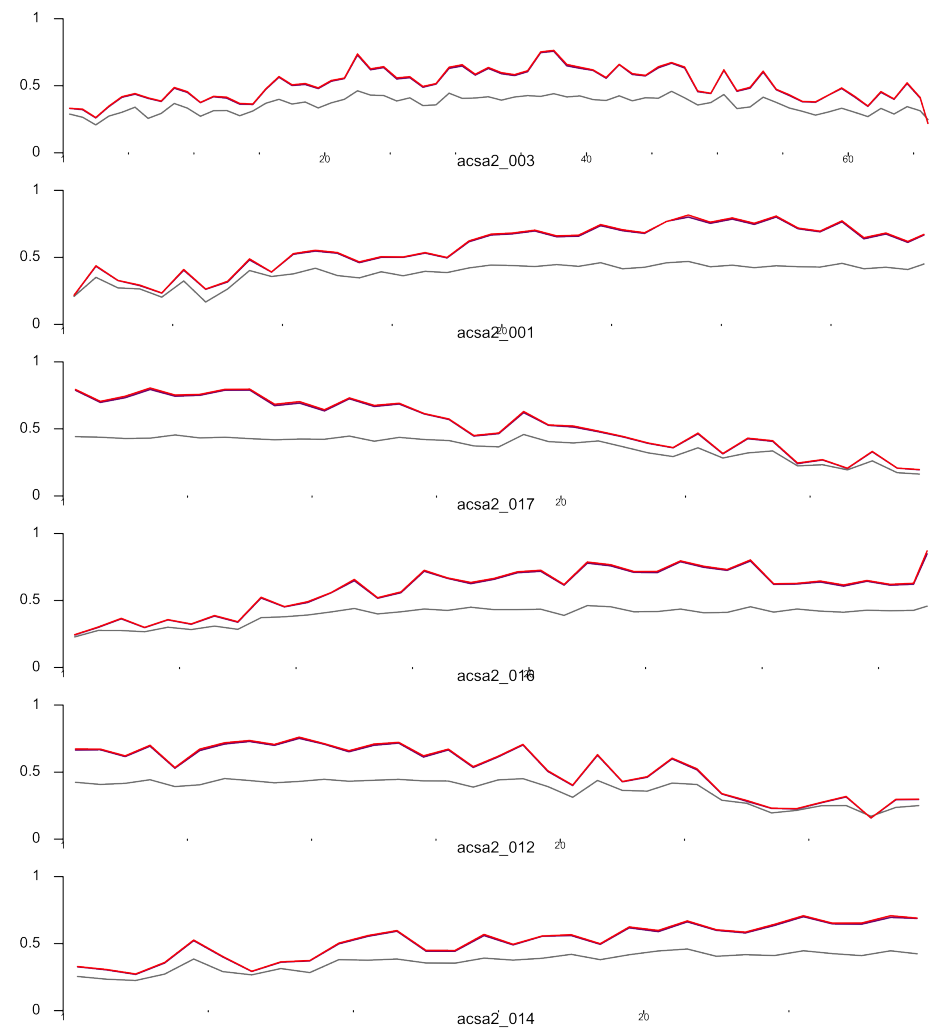

Supplement: Supplementary file 2 — Figure S2. Distribution of CHG methylation by subcontext across all chromosomes (1 Mbp window every 1 Mbp) in new and original Acer negundo (acne) and Acer saccharum (acsa) genomes. [file EVA-17-e13669-s004.pdf]

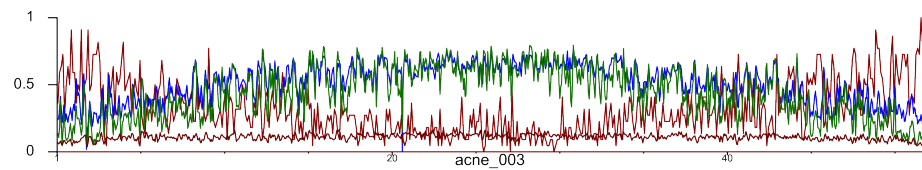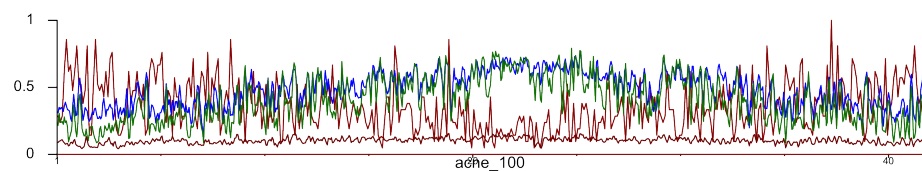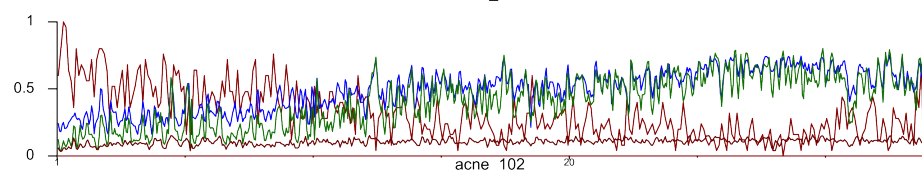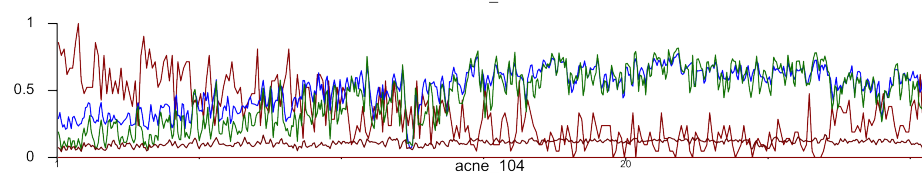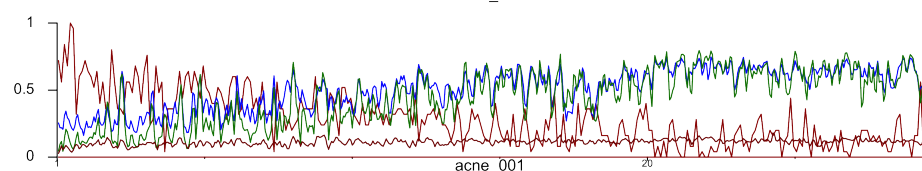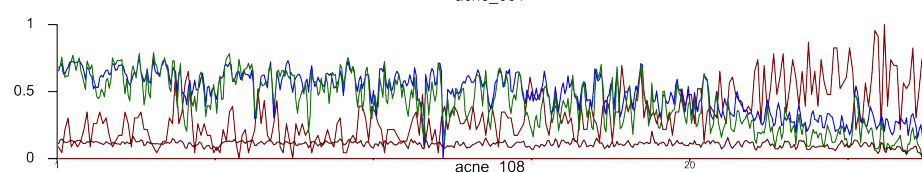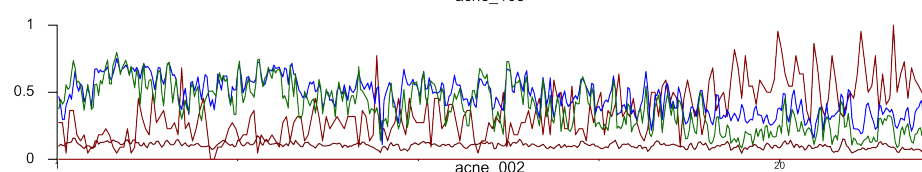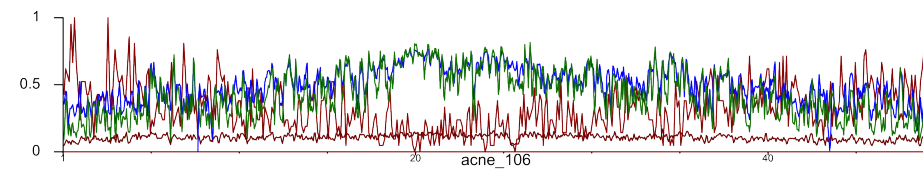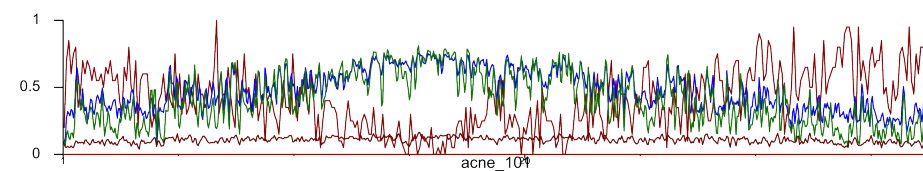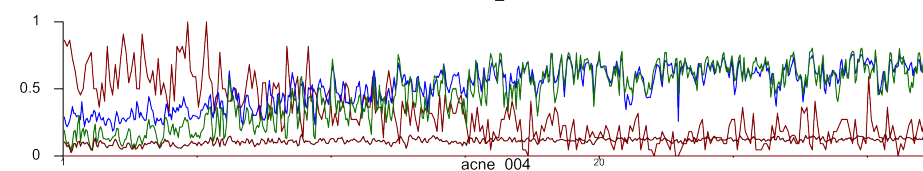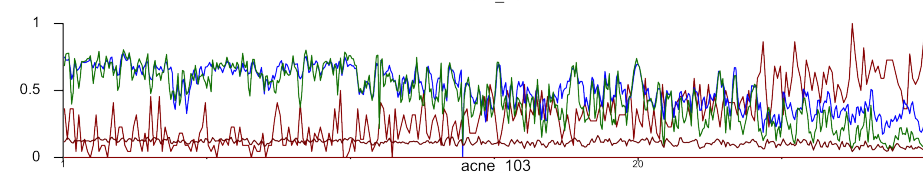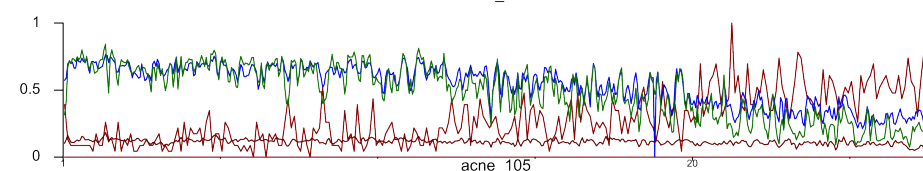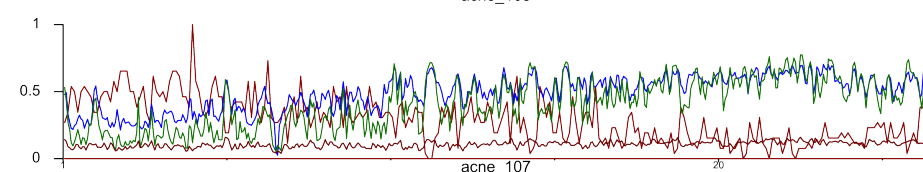

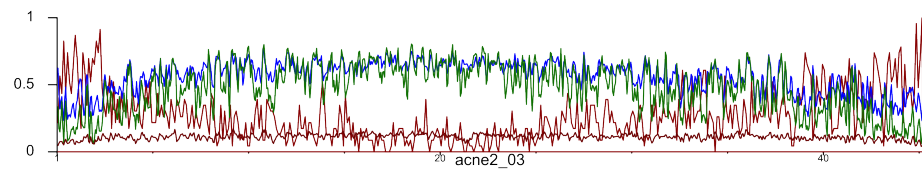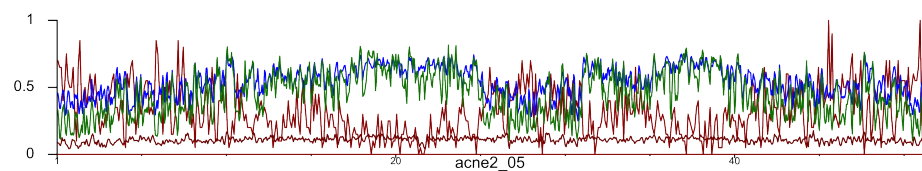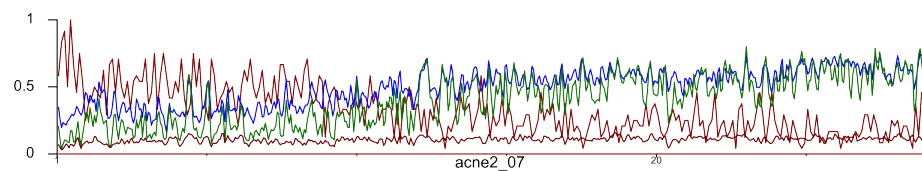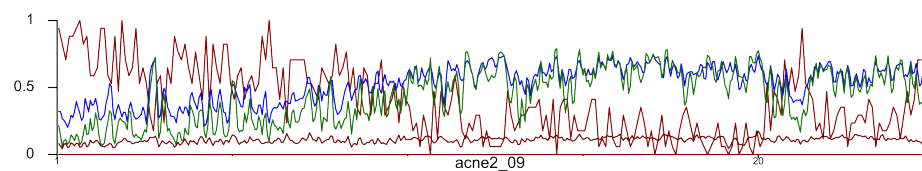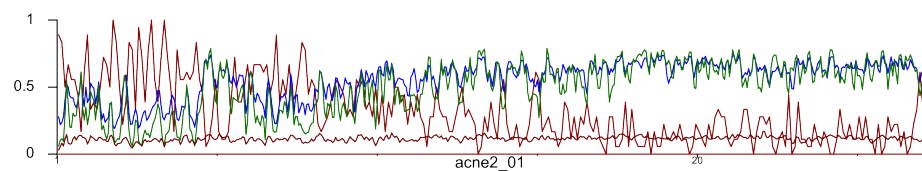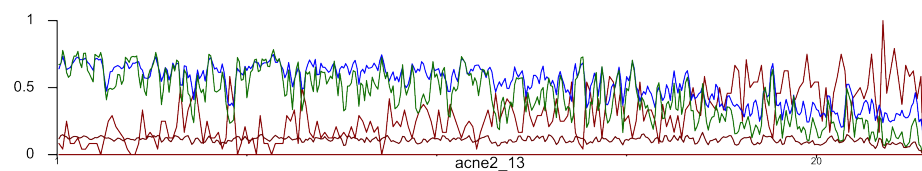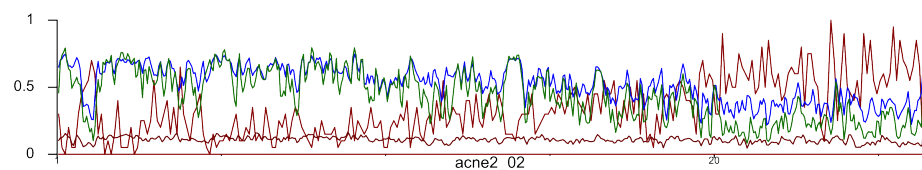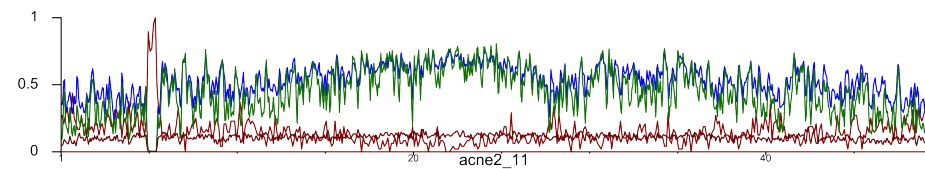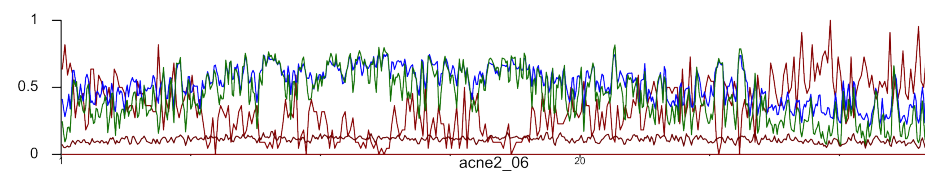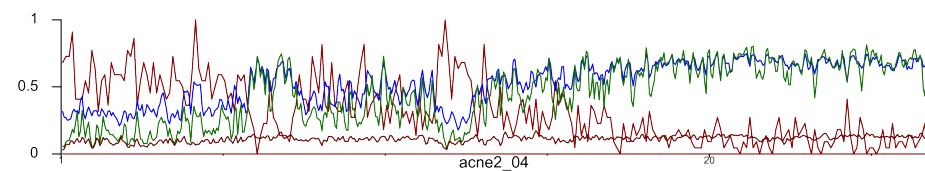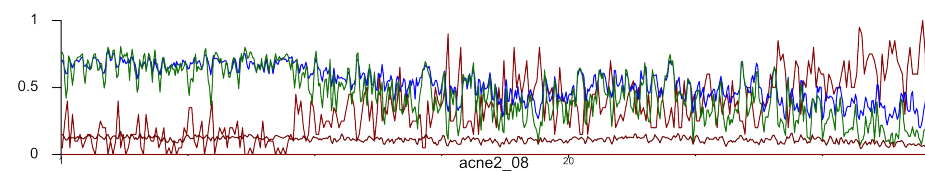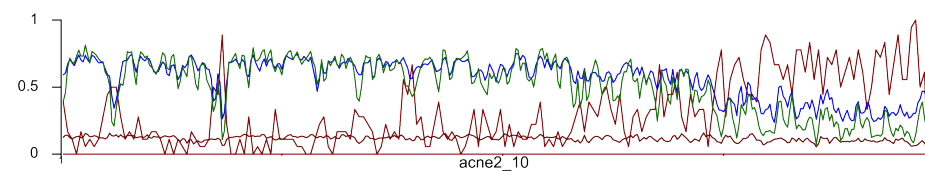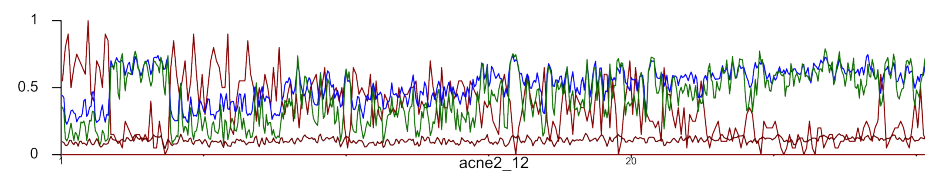

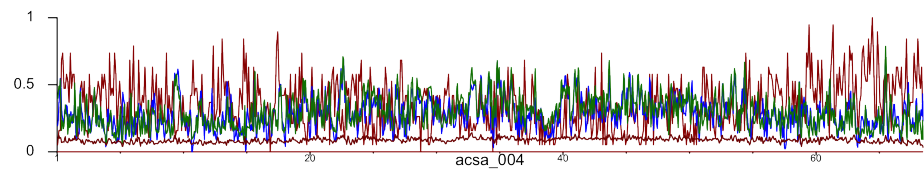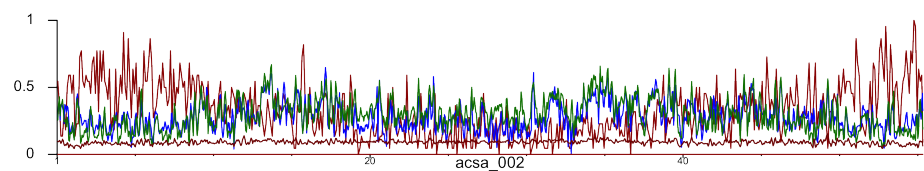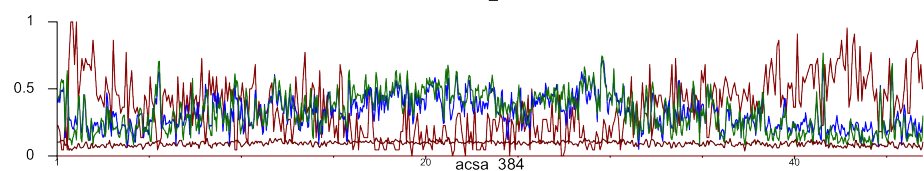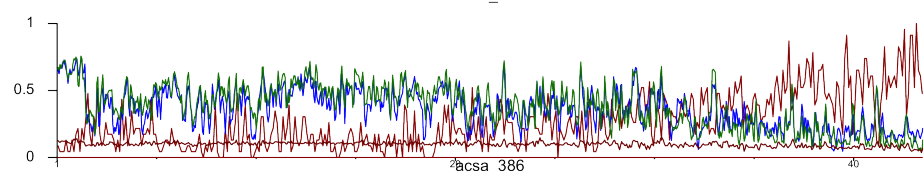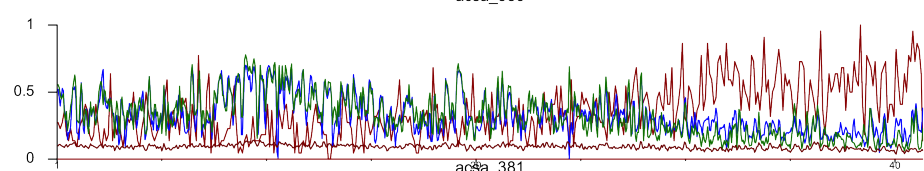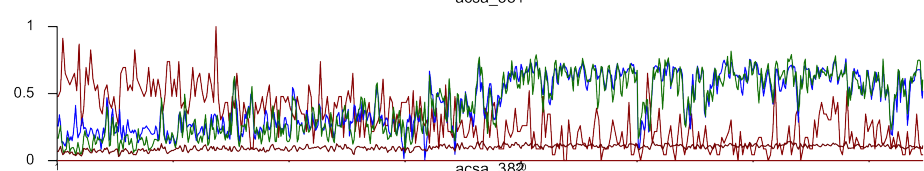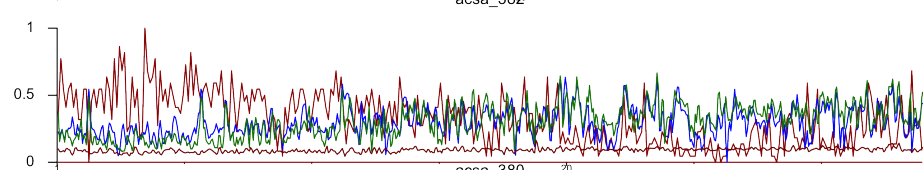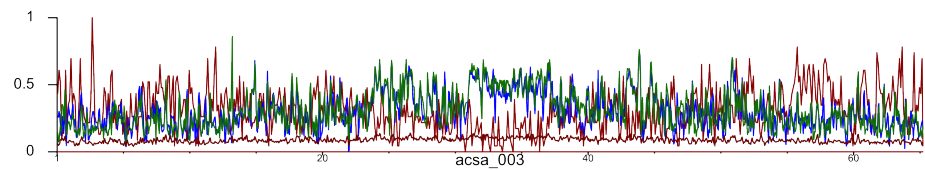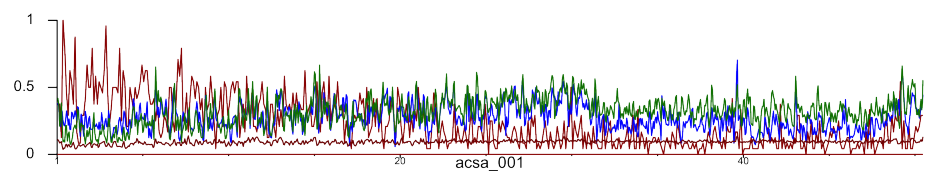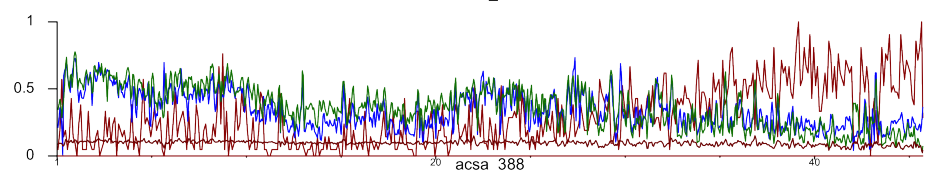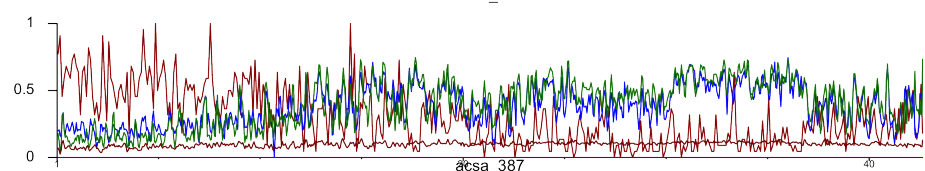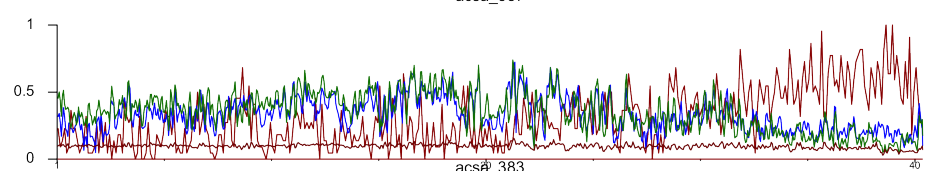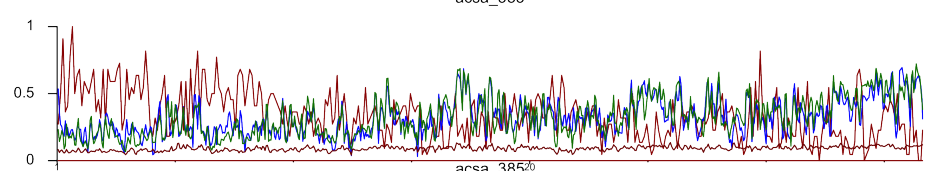

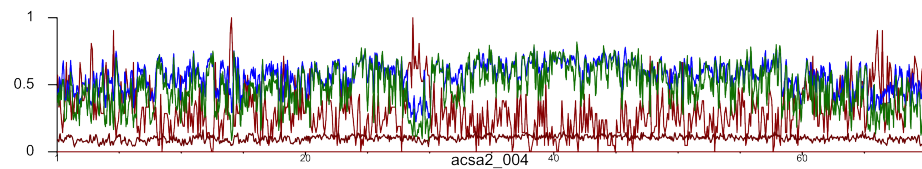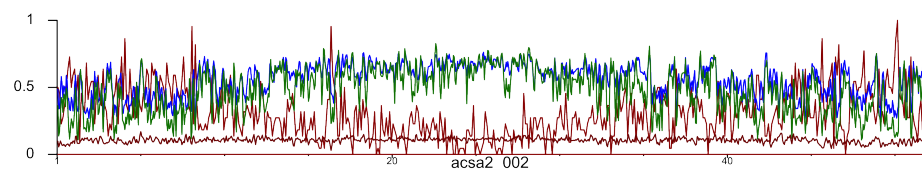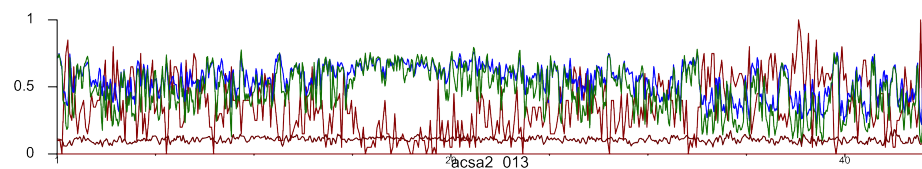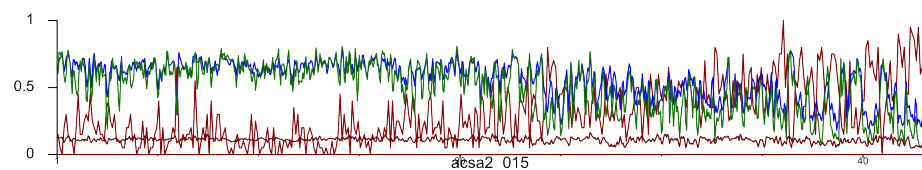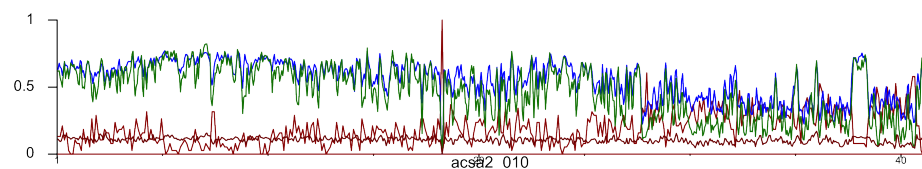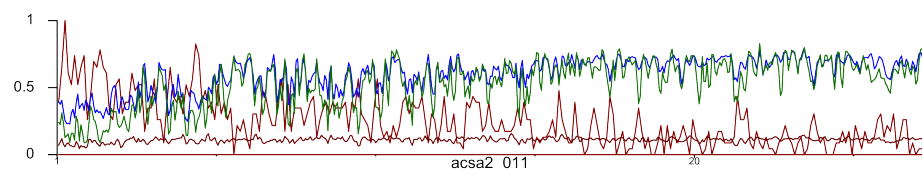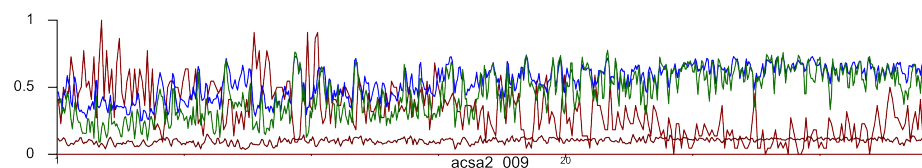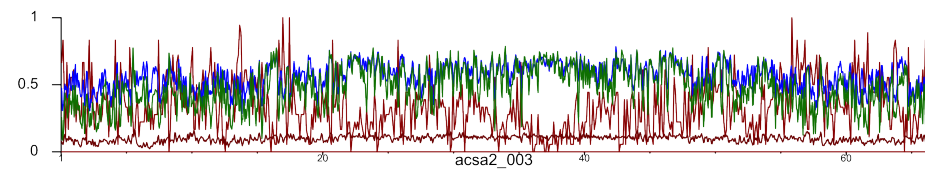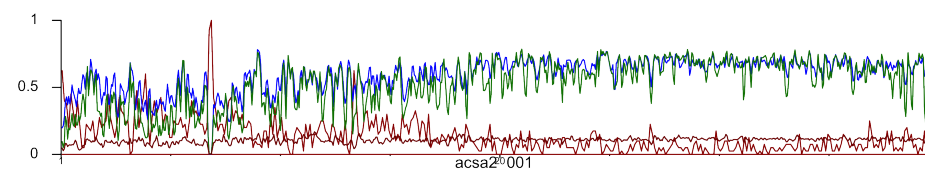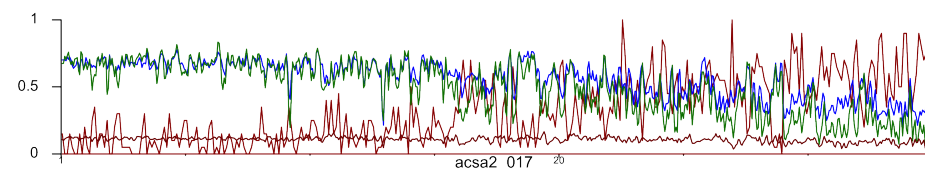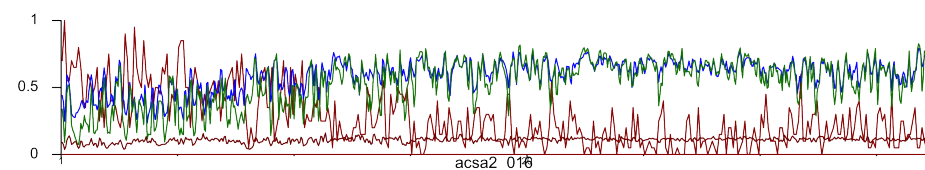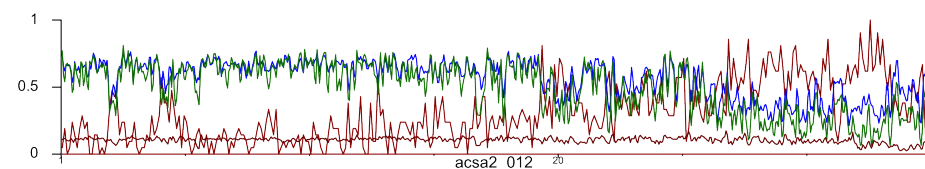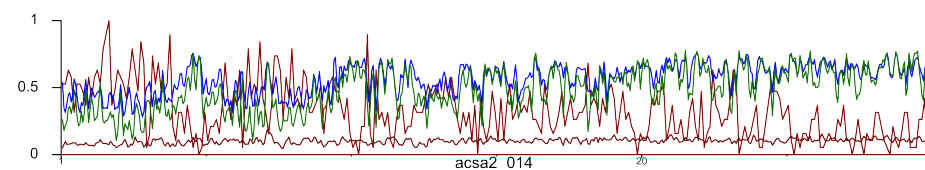

Supplement: Supplementary file 3 — Figure S3. Gene densities (red) with CG (blue), CHG (green), and CHH (maroon) contexts in 100 Kb windows across new and original Acer negundo (acne) and Acer saccharum (acsa) genomes. [file EVA-17-e13669-s006.pdf]

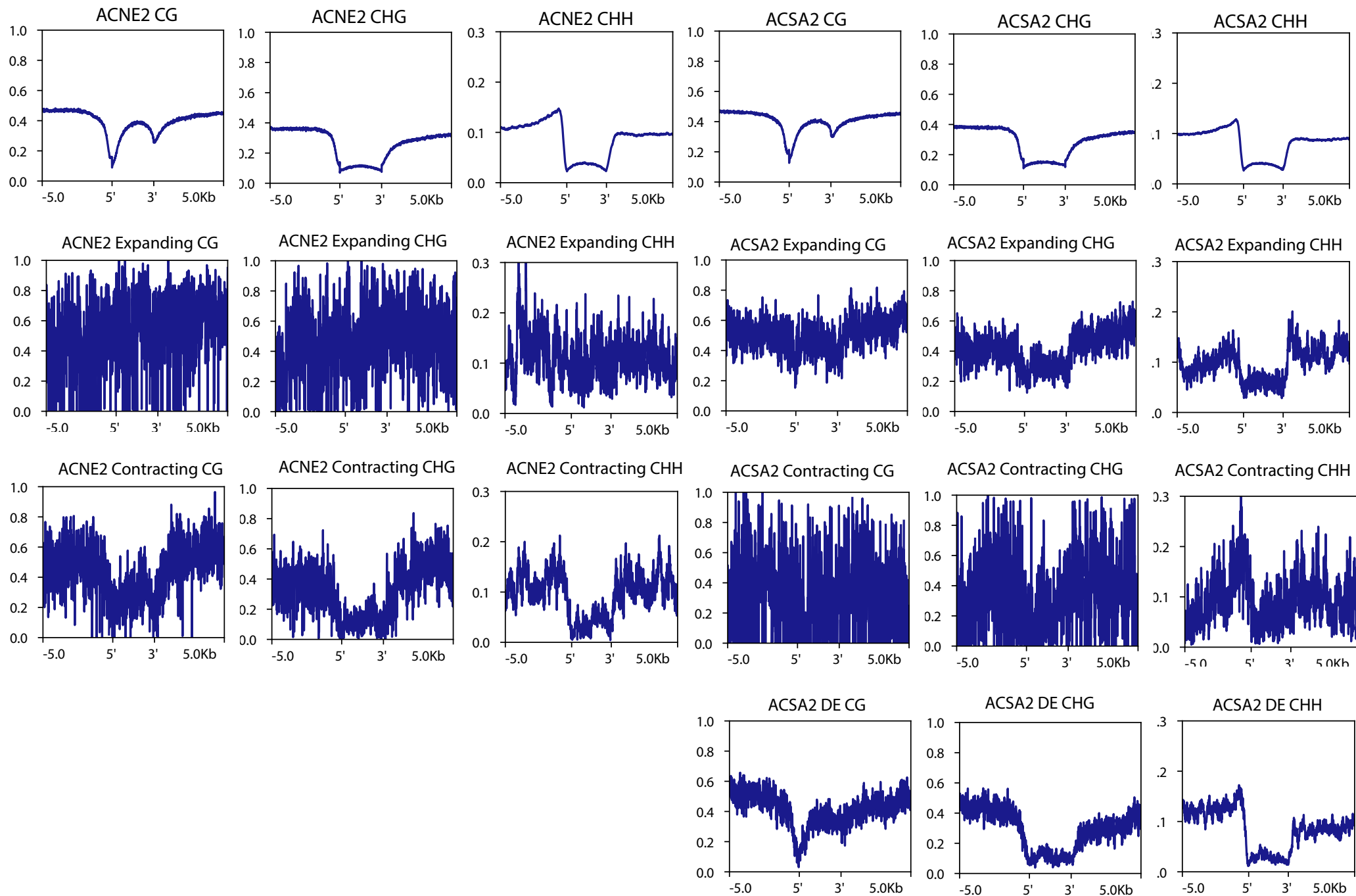

Supplement: Supplementary file 4 — Figure S4. (a) Methylation frequency distribution across protein coding regions, 5′ to 3′, shown by assembly and sequence context for new Acer negundo (acne2) and Acer saccharum (acsa2) genomes. Top row includes results from whole‐genome mean, second row is genes from rapidly expanding gene families, third row is genes from rapidly contracting families. Bottom row shows methylation frequency distribution across 240 (of the original 245) genes differentially expressed in response to calcium and aluminum treatments in stem as seen in McEvoy et al. (2021). [file EVA-17-e13669-s005.pdf]

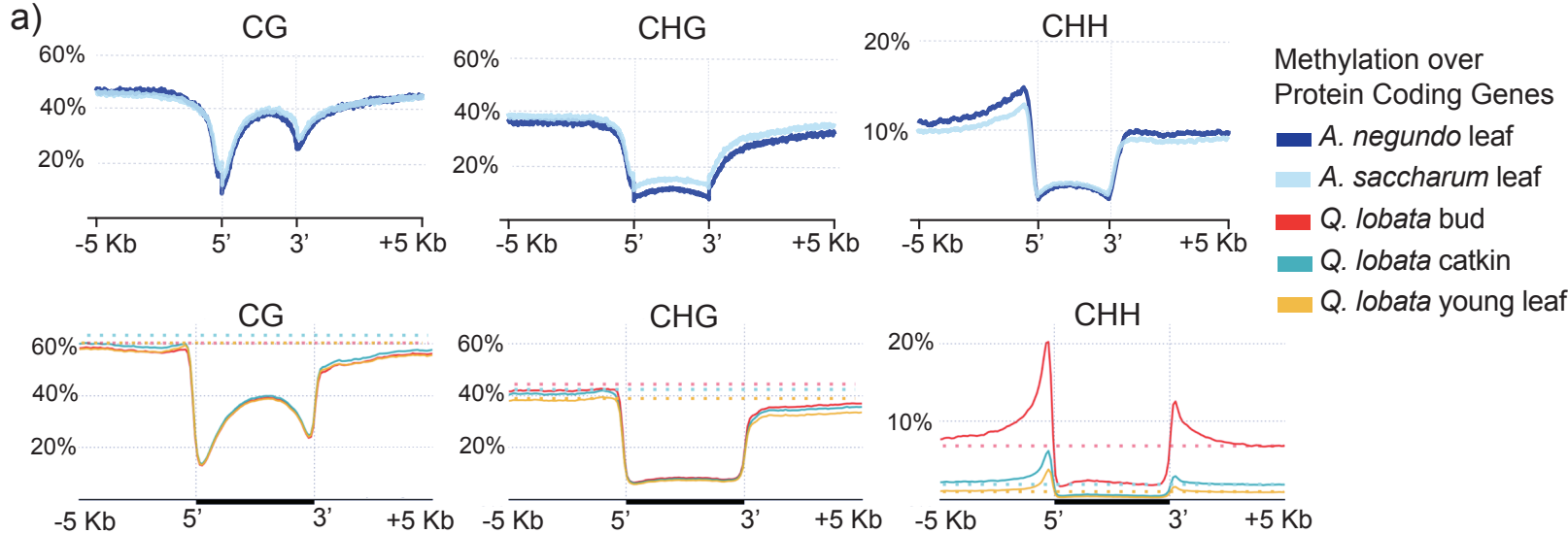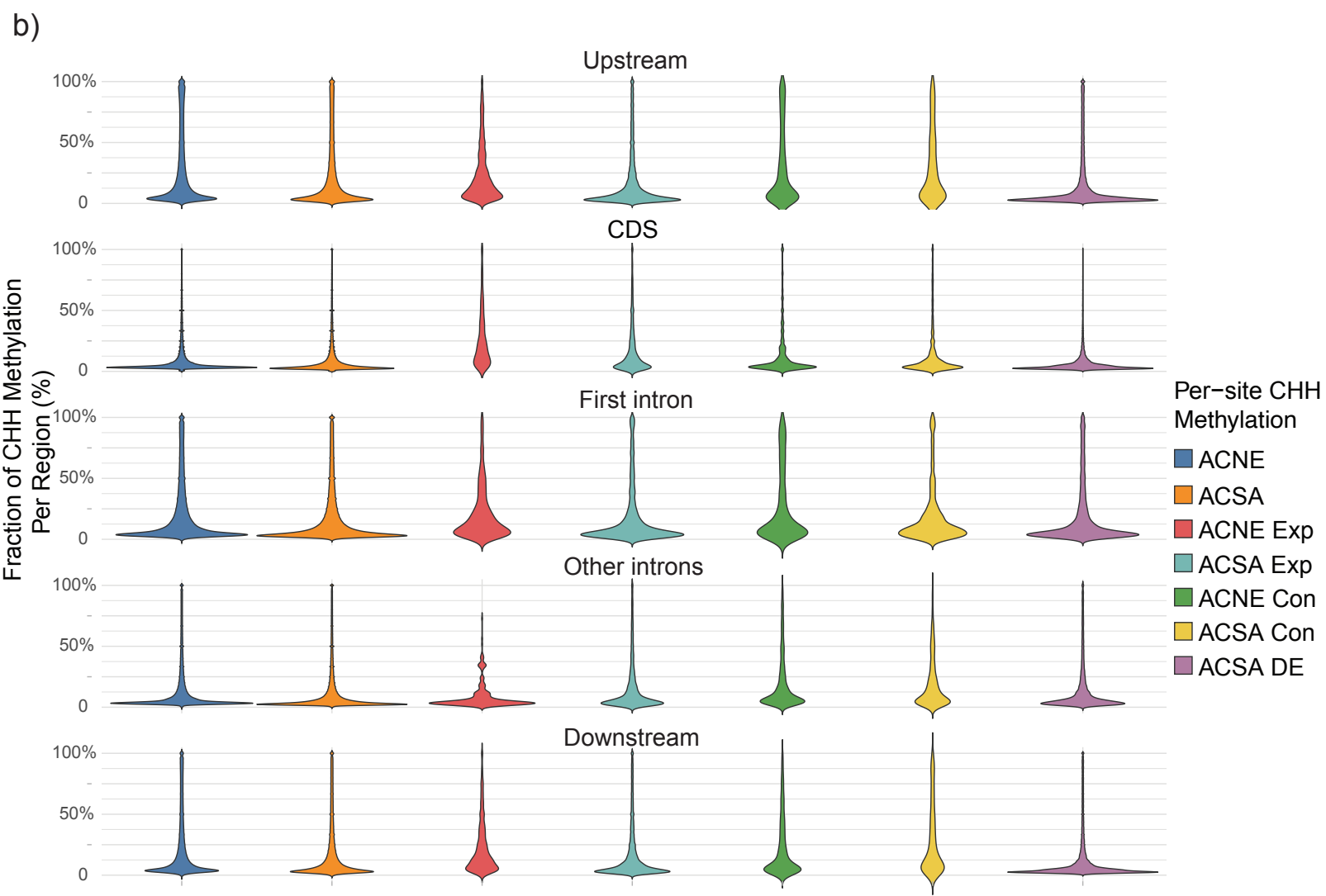

Supplement: Supplementary file 5 — Figure S5. Chromosome‐level distribution of gene density, LTR repeat families and Copia subfamilies, methylation by context, CHH methylation subcontexts, gene family dynamics, and gene expression results for select chromosomes. New Acer negundo (acne2) and Acer saccharum (acsa2) are shown. Genes from expanded gene families are shown as lime green vertical bars, and those from contracted, as purple bars. Acer saccharum gene expression results (red and blue dots) are from aluminum and calcium treatments at Hubbard Brook Experimental Forest as detailed in McEvoy et al. (2021). X‐axis indicates log2 fold change, while dot size represents the p‐adjusted value. [file EVA-17-e13669-s001.pdf]
